# RTP801 interacts with the tRNA ligase complex and dysregulates its RNA ligase activity in Alzheimer’s disease

**DOI:** 10.1093/nar/gkae776

**Published:** 2024-09-12

**Authors:** Genís Campoy-Campos, Julia Solana-Balaguer, Anna Guisado-Corcoll, Almudena Chicote-González, Pol Garcia-Segura, Leticia Pérez-Sisqués, Adrian Gabriel Torres, Mercè Canal, Laura Molina-Porcel, Joaquín Fernández-Irigoyen, Enrique Santamaria, Lluís Ribas de Pouplana, Jordi Alberch, Eulàlia Martí, Albert Giralt, Esther Pérez-Navarro, Cristina Malagelada

**Affiliations:** Departament de Biomedicina, Institut de Neurociències, Universitat de Barcelona, Barcelona 08036, Catalonia, Spain; Centro de Investigación Biomédica en Red sobre Enfermedades Neurodegenerativas (CIBERNED), Madrid 28029, Spain; Departament de Biomedicina, Institut de Neurociències, Universitat de Barcelona, Barcelona 08036, Catalonia, Spain; Centro de Investigación Biomédica en Red sobre Enfermedades Neurodegenerativas (CIBERNED), Madrid 28029, Spain; Departament de Biomedicina, Institut de Neurociències, Universitat de Barcelona, Barcelona 08036, Catalonia, Spain; Centro de Investigación Biomédica en Red sobre Enfermedades Neurodegenerativas (CIBERNED), Madrid 28029, Spain; Institut d’Investigacions Biomèdiques August Pi i Sunyer (IDIBAPS), Barcelona 08036 Catalonia, Spain; Departament de Biomedicina, Institut de Neurociències, Universitat de Barcelona, Barcelona 08036, Catalonia, Spain; Centro de Investigación Biomédica en Red sobre Enfermedades Neurodegenerativas (CIBERNED), Madrid 28029, Spain; Departament de Biomedicina, Institut de Neurociències, Universitat de Barcelona, Barcelona 08036, Catalonia, Spain; Centro de Investigación Biomédica en Red sobre Enfermedades Neurodegenerativas (CIBERNED), Madrid 28029, Spain; Departament de Biomedicina, Institut de Neurociències, Universitat de Barcelona, Barcelona 08036, Catalonia, Spain; Centro de Investigación Biomédica en Red sobre Enfermedades Neurodegenerativas (CIBERNED), Madrid 28029, Spain; Institut de Recerca Biomèdica (IRB Barcelona), Barcelona 08028, Catalonia, Spain; Barcelona Institute of Science and Technology (BIST), Barcelona 08028, Catalonia, Spain; Departament de Biomedicina, Institut de Neurociències, Universitat de Barcelona, Barcelona 08036, Catalonia, Spain; Centro de Investigación Biomédica en Red sobre Enfermedades Neurodegenerativas (CIBERNED), Madrid 28029, Spain; Alzheimer’s Disease and Other Cognitive Disorders Unit, Neurology Service, Hospital Clínic, Fundació de Recerca Clínic Barcelona-Institut d’Investigacions Biomèdiques August Pi i Sunyer (FRCB-IDIBAPS), University of Barcelona, Barcelona 08036, Catalonia, Spain; Neurological Tissue Bank, Biobank-Hospital Clínic-FRCB-IDIBAPS, Barcelona 08036, Catalonia, Spain; Proteored-ISCIII, Proteomics Unit, Navarrabiomed, Departamento de Salud, UPNA, IdiSNA, Pamplona 31008, Spain; Proteored-ISCIII, Proteomics Unit, Navarrabiomed, Departamento de Salud, UPNA, IdiSNA, Pamplona 31008, Spain; Institut de Recerca Biomèdica (IRB Barcelona), Barcelona 08028, Catalonia, Spain; Barcelona Institute of Science and Technology (BIST), Barcelona 08028, Catalonia, Spain; Institució Catalana de Recerca i Estudis Avançats (ICREA), Barcelona 08010, Catalonia, Spain; Departament de Biomedicina, Institut de Neurociències, Universitat de Barcelona, Barcelona 08036, Catalonia, Spain; Centro de Investigación Biomédica en Red sobre Enfermedades Neurodegenerativas (CIBERNED), Madrid 28029, Spain; Institut d’Investigacions Biomèdiques August Pi i Sunyer (IDIBAPS), Barcelona 08036 Catalonia, Spain; Faculty of Medicine and Health Science, Production and Validation Center of Advanced Therapies (Creatio), Universitat de Barcelona, Barcelona 08036, Catalonia, Spain; Departament de Biomedicina, Institut de Neurociències, Universitat de Barcelona, Barcelona 08036, Catalonia, Spain; Departament de Biomedicina, Institut de Neurociències, Universitat de Barcelona, Barcelona 08036, Catalonia, Spain; Centro de Investigación Biomédica en Red sobre Enfermedades Neurodegenerativas (CIBERNED), Madrid 28029, Spain; Institut d’Investigacions Biomèdiques August Pi i Sunyer (IDIBAPS), Barcelona 08036 Catalonia, Spain; Faculty of Medicine and Health Science, Production and Validation Center of Advanced Therapies (Creatio), Universitat de Barcelona, Barcelona 08036, Catalonia, Spain; Departament de Biomedicina, Institut de Neurociències, Universitat de Barcelona, Barcelona 08036, Catalonia, Spain; Centro de Investigación Biomédica en Red sobre Enfermedades Neurodegenerativas (CIBERNED), Madrid 28029, Spain; Institut d’Investigacions Biomèdiques August Pi i Sunyer (IDIBAPS), Barcelona 08036 Catalonia, Spain; Departament de Biomedicina, Institut de Neurociències, Universitat de Barcelona, Barcelona 08036, Catalonia, Spain; Centro de Investigación Biomédica en Red sobre Enfermedades Neurodegenerativas (CIBERNED), Madrid 28029, Spain

## Abstract

RTP801/REDD1 is a stress-responsive protein overexpressed in neurodegenerative diseases such as Alzheimer’s disease (AD) that contributes to cognitive deficits and neuroinflammation. Here, we found that RTP801 interacts with HSPC117, DDX1 and CGI-99, three members of the tRNA ligase complex (tRNA-LC), which ligates the excised exons of intron-containing tRNAs and the mRNA exons of the transcription factor XBP1 during the unfolded protein response (UPR). We also found that RTP801 modulates the mRNA ligase activity of the complex *in vitro* since RTP801 knockdown promoted *XBP1* splicing and the expression of its transcriptional target, *SEC24D*. Conversely, RTP801 overexpression inhibited the splicing of *XBP1*. Similarly, in human AD postmortem hippocampal samples, where RTP801 is upregulated, we found that XBP1 splicing was dramatically decreased. In the 5xFAD mouse model of AD, silencing RTP801 expression in hippocampal neurons promoted *Xbp1* splicing and prevented the accumulation of intron-containing pre-tRNAs. Finally, the tRNA-enriched fraction obtained from 5xFAD mice promoted abnormal dendritic arborization in cultured hippocampal neurons, and RTP801 silencing in the source neurons prevented this phenotype. Altogether, these results show that elevated RTP801 impairs RNA processing *in vitro* and *in vivo* in the context of AD and suggest that RTP801 inhibition could be a promising therapeutic approach.

## Graphical Abstract

**Graphical Abstract:**
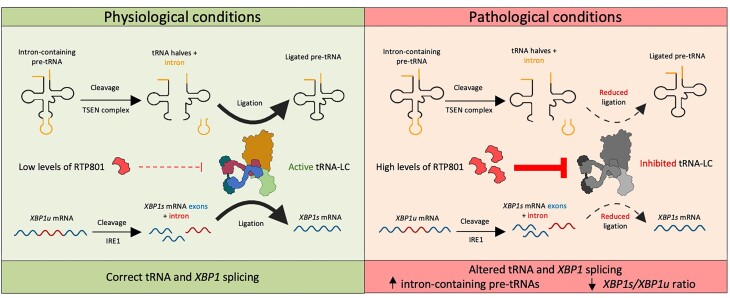

## Introduction

Alzheimer’s disease (AD) is the most prevalent form of dementia, marked by a progressive decline in cognitive abilities and executive function, as well as by memory loss. It also affects language, and patients may present emotional and psychiatric symptoms. AD is characterized by the presence of extracellular amyloid-β plaques and intracellular tangles of hyperphosphorylated tau. Their accumulation disrupts synaptic functioning, triggers neuroinflammation and leads to neuronal death, resulting in brain atrophy and gliosis. The exact mechanisms by which these events take place are not yet elucidated, but RNA translation impairment could contribute to the pathogenesis of the disease (1,2).

RTP801, also known as REDD1, is a stress-induced protein ubiquitously expressed at low levels (3,4). The transcription of its coding gene *DDIT4* increases with exposure to amyloid-β (5), mutant huntingtin (6) or 6-hydroxydopamine (7), among others. Indeed, increased levels of RTP801 have been found in the brains of patients with neurodegenerative disorders such as AD (8), Huntington’s (HD) (6,9) and Parkinson’s diseases (7,10,11). On the other hand, RTP801 knockout mice show improved motor learning (12) and are more resilient to stress-induced synaptic and behavioral deficits (13). To date, the main described function of RTP801 is to negatively regulate mechanistic target of rapamycin (mTOR) and Akt, kinases that are essential for translation and survival, respectively (14). In this line, RTP801 also modulates neuroinflammation in AD and HD (8,9) and mediates transneuronal toxicity *in vitro* via extracellular vesicles (EVs) (15), processes that mostly depend on *de novo* protein synthesis of inflammasome and pro-apoptotic effectors.

As aforementioned, translation can be controlled not only by the activity of mTOR but also by modulating tRNA (16) and mRNA (17) levels. Thus, the processing of both mRNAs and tRNAs is crucial for the regulation of protein synthesis. In this sense, the tRNA ligase complex (tRNA-LC) is a pentameric complex that catalyzes the ligation of tRNA (18) and mRNA (19) exons during splicing. The essential subunit of the complex, which constitutes its core (20) and presents the ligase activity is called HSPC117 (18). The other components of this complex are the RNA helicase DDX1, and the adaptor proteins CGI-99, ASW and FAM98B (18).

As its name implies, the tRNA-LC is responsible for the ligation of tRNA exons during tRNA splicing. tRNA genes are transcribed by RNA polymerase III to generate precursor tRNA molecules (pre-tRNAs), which are processed to obtain mature tRNAs (21). All pre-tRNAs contain 5′ leader and 3′ trailer sequences but only a small percentage of them have an intron (∼7% in humans and ∼5% in mice) (22,23) (http://gtrnadb.ucsc.edu/genomes/eukaryota/Mmusc39/). In vertebrates, tRNA processing begins in the nucleus and includes intron splicing (if present), removal of the 5′ leader and the 3′ trailer, and addition of a cytosine-cytosine-adenine (CCA) trinucleotide to the 3′ end. tRNAs are then exported to the cytoplasm where they are aminoacylated by aminoacyl synthetases. Throughout the processing, many posttranscriptional modifications are introduced to the pre-tRNAs, which are essential for their proper function (24). The splicing of intron-containing pre-tRNAs consists in two steps: cleavage and ligation. In mammals, cleavage is performed by the tRNA splicing endonuclease (TSEN) complex in collaboration with the RNA kinase Clp1 (25,26), which results in the production of 2 exons and 1 intron. The ligation is performed by the tRNA-LC, and specifically, by the ligase HSPC117 (18).

Moreover, the tRNA-LC participates in the unconventional splicing of *XBP1* mRNA during the unfolded protein response (UPR) (19). Briefly, *XBP1* mRNA undergoes canonical splicing in the nucleus but retains a short, 26-nucleotide intron. This mRNA (*XBP1u*) is translated, producing a protein involved in various biological pathways (27). However, the accumulation of unfolded or misfolded proteins in the endoplasmic reticulum (ER) activates the UPR, which leads to the cleavage of *XBP1* mRNA in the cytoplasm by IRE1 and the subsequent ligation by the tRNA-LC. The spliced mRNA (*XBP1s*) is translated generating a transcription factor that helps to restore ER homeostasis (28).

Here, we investigated whether RTP801 is a novel regulator of the tRNA-LC and whether RTP801 upregulation in a pathological context affects its mRNA and tRNA ligase activity. Using *in vitro* models and the 5xFAD mouse model of AD, along with human AD postmortem samples, we can confirm that RTP801 interacts with the complex and interferes with its RNA ligase activity to promote a pathological phenotype.

## Materials and methods

### Human postmortem samples

Postmortem hippocampal samples from AD patients and age-matched control individuals were acquired from the Neurological Tissue Bank, (Biobank-Hospital Clínic-FRCB-IDIBAPS, Barcelona). All brain tissue samples were obtained from patients after they or their legal representatives gave written informed consent for the use of their brain tissue and medical records for research purposes, as approved by the Ethics Committee of the Brain Bank institution, in accordance with the Declaration of Helsinki. The neuropathological examination was performed according to standardized protocols at the Neurological Tissue Bank of the IDIBAPS Biobank. Briefly, half of the brain was dissected in the fresh state, then frozen and stored at −80°C, while the remaining half was fixed in formaldehyde solution for three weeks. For histological evaluation, 5-μm-thick paraffin-embedded sections from at least 25 representative brain regions were stained, and immunohistochemistry was performed (see (29) for more information). Supplementary Table S1 contains case information.

### Animal models

The transgenic mouse line 5xFAD (MMRRC catalog #034840-JAX, RRID:MMRRC_034840-JAX) was utilized for this investigation. 5xFAD mice overexpress the 695-amino acid isoform of the human amyloid precursor protein (APP695) carrying the Swedish, London and Florida mutations under the control of the murine Thy-1 promoter. Additionally, 5xFAD mice express human presenilin-1 (PSEN-1) with the M146L/L286V mutation, also under the control of the Thy-1 promoter (30). All animals were housed in colony rooms with a 12:12-h light/dark cycle, maintained at 19–22°C and 40–60% humidity, with unlimited access to food and water. All the experimental animals were male, and they were used from 6 months of age onward. All procedures were in accordance with the Guide for the Care and Use of Laboratory Animals (NIH Publication No. 85–23, 1985 Revision), the European Community Guidelines, and Spanish guidelines (RD53/2013) and they were approved by the local ethical committee (University of Barcelona, 55/21 and Generalitat de Catalunya, 11559).

### Rat primary cortical cultures

Rat primary cortical cultures were prepared by dissecting out the cortex from embryonic day 18 (E18) Sprague‐Dawley rats as previously described (6). Cells were seeded at a density of 750 cells/mm^2^ on plastic plates coated with 0.25 mg/ml poly-L-lysine (from Merck). Neurons were maintained in Neurobasal medium supplemented with serum-free B27 (1:50), 2 mM GlutaMAX, and penicillin/streptomycin (all from Thermo Fisher Scientific). Cell cultures were kept in a 5% CO_2_ atmosphere at 37°C. All experiments were performed at 13–14 days *in vitro* (DIV).

### Mouse primary hippocampal cultures

Mouse primary hippocampal cultures were prepared by dissecting out the hippocampus from E18 B6CBA WT mice. The tissue was dissociated with 0.25% trypsin (from Thermo Fisher Scientific) for 15 min, followed by a mechanical dissociation with rounded-end glass Pasteur pipettes. Cells were seeded at a density of 400 cells/mm^2^ onto autoclaved 12 mm glass coverslips pre-coated with 0.1 mg/ml poly-D-lysine (from Merck). Neurons were maintained in supplemented Neurobasal medium and kept in a 5% CO_2_ atmosphere at 37°C. Experiments were performed at 13–14 DIV.

### HEK293 cells culture

Human Embryonic Kidney 293 (HEK293) is a cell line originally derived from human embryonic kidney cells. In this study, HEK293 cells were cultured in plastic plates with Dulbecco’s modified Eagle’s medium (DMEM) supplemented with 10% fetal bovine serum and 1% penicillin/streptomycin (all from Thermo Fisher Scientific). Cells were kept in a 5% CO_2_ atmosphere at 37°C and were reseeded when confluent.

### Stereotaxic surgery and AAV transduction

Six-month-old wild-type (WT) and 5xFAD mice received bilateral hippocampal injections of adeno-associated viruses (AAVs) to genetically silence RTP801 in neurons, as described in (8). Summarizing, mice were injected in the CA1 and in the dentate gyrus with neuron-targeted rAAV2/8-H1-shControl-RSV-GFP (1.2 × 10^13^ genome copies [GCs]) or rAAV2/8-H1-shRTP801-RSV-GFP (1.07 × 10^13^ GCs) (Supplementary Table S2). After 2 h of careful monitoring, mice were returned to their cage for 4 weeks and were then euthanized for biochemical analysis.

### Immunoprecipitation

In order to detect weak or transient interactions, HEK293 cells were treated for 2 h with dithiobis(succinimidyl propionate) (DSP), a protein cross-linker that forms stable amide bonds between molecules and following manufacturer’s instructions (Thermo Fisher Scientific). Cells were then lysed with Cell Lysis Buffer (from Cell Signaling Technology) supplemented with 1% phenylmethylsulphonyl (PMSF, from Merck) and centrifuged at 14000 × *g*for 10 min to remove debris. The total protein concentration of the supernatant was quantified using the Bio-Rad Protein Assay. Protein A-agarose beads (from Santa Cruz Biotechnology) were thoroughly washed with 3-((3-cholamidopropyl) dimethylammonium)-1-propanesulfonate (CHAPS) buffer (50 mM Tris pH = 7.4, 150 mM NaCl, 10 mM MgCl_2_, 0.4% CHAPS) (all from Merck). Then, samples, beads and antibodies of interest (Supplementary Table S3) were mixed and incubated overnight on rotation at 4°C. Numerous negative controls were included to ensure specificity: first, beads and samples were incubated without antibodies; second, rabbit normal immunoglobulins (IgG) (Merck) were added instead of the appropriated antibody; and third, lysis buffer was incubated instead of samples. The next day, the mixtures were washed four times with CHAPS buffer supplemented with protease inhibitor (cOmplete™ Mini Protease Inhibitor Cocktail), phosphatase inhibitor (PhosSTOP™), and 1% PMSF (all from Merck). Reducing loading buffer (from Thermo Fisher Scientific) was added to the mixtures, which were heated at 96°C for 5 min for protein denaturation. Mixtures were then centrifuged 20 s at 14000 × *g* and supernatant was stored at −20°C until western blot (WB) was performed.

### Western blot

Human hippocampal postmortem tissue or dissected mouse hippocampi were homogenized in lysis buffer supplemented with 1% PMSF. Cell cultures were lysed in the same way. Samples were centrifuged and protein content was assessed as previously stated. Samples (20 μg per lane) were resolved in 4–12% polyacrylamide gels and the following steps were performed as previously described in (8). The primary antibodies used in this study are summarized in Supplementary Table S3. Note that the antibody used to detect XBP1s (marked by arrows in the figures) and XBP1u (Abcam #ab37152) also detected an unspecific band just below the 57 kDa XBP1s.

The WB membranes used to prepare the figures with the representative bands indicated with red rectangles are shown in Supplementary Figure S6.

### In-gel tryptic digestion and mass spectrometry

RTP801 was immunoprecipitated as previously described and the resulting immunocomplexes were separated by polyacrylamide gel electrophoresis. Gels were incubated twice for 30 min with fix solution (50% methanol, 7% acetic acid) and stained overnight at 4°C with Sypro® Ruby Protein Gel Stain (from Thermo Fisher Scientific). Then, gels were washed 30 min with washing solution (10% methanol and 7% acetic acid) and 10 min with double distilled water. A blue light transilluminator was used to view and cut the bands of interest, which were distained with 50 mM ammonium bicarbonate in 50% acetonitrile. Then, proteins were reduced with 10 mM dithiothreitol in 100 mM ammonium bicarbonate and alkylated with 55 mM iodoacetamide (IAA) in 100 mM ammonium bicarbonate. In-gel protein digestion was carried out with 10 ng trypsin (Promega) in 50 mM ammonium bicarbonate for 12 h at 37°C as described in (31). The resulting peptides were extracted in 2% formic acid in 2% acetonitrile and were analyzed by liquid chromatography-tandem mass spectrometry (LC-MS/MS) by the Proteomics Unit of Navarrabiomed. Concretely, peptides were separated by reverse phase chromatography using an Eksigent nanoLC ultra 2D pump fitted with a 75 μm ID column. Previously, samples were loaded into a 0.5 cm length 100 μm ID precolumn for desalting and concentration. Mobile phases consisted of 0.1% formic acid in water (buffer A) and 0.1% formic acid in acetonitrile (buffer B). Column was equilibrated in 95% buffer B during 10 min and in 5% buffer B for 10 min. Elution was carried out with a 2-step gradient of 60 min: from 5% buffer B to 25% buffer B in 50 min and from 25% buffer B to 40% buffer B in 10 min. During all process, precolumn and column were in line and the flow was maintained at 300 nl/min. Eluting peptides were analyzed using an AB Sciex 5600 Triple-TOF system. Data were acquired upon a survey scan performed in a mass range between 350 and 1250 *m/z* during 250 ms. Top 30 peaks were selected for further fragmentation. Minimum accumulation time for MS/MS was set up at 100 ms giving a total cycle time of 3.8 s. Product ions were scanned in a mass range from 230 to 1500 *m/z* and were excluded for further fragmentation for 15 s. After MS/MS analysis, data files were processed using ProteinPilot™ 4.5 software (Sciex) which uses the algorithm Paragon™ (v.4.0.0.0) for database search and Progroup™ for data grouping and searched against Uniprot Human database. The search parameters allowed for cysteine modification by IAA and default biological modifications programmed in the algorithm.

### Plasmid transfection

To modulate the mRNA and protein levels of RTP801 in HEK293 cells, cells were transfected with shRNA or RTP801-overexpressing vectors, using the polymer polyethyleneimine (PEI, from CliniSciences) and following manufacturer’s instructions. HSPC117 was knocked down in HEK293 cells using the same procedure. Plasmids used are summarized in Supplementary Table S2. In brief, PEI was diluted in Milli-Q water to 1 mg/ml, and it was filtered through a 0.22 μm filter. It was then mixed with the respective DNA vector (4 μg of PEI per each μg of DNA) and non-supplemented DMEM medium for 20 min at room temperature (RT). After that, the medium of HEK293 cells was replaced by the mixture and it was incubated 4 h in a 5% CO_2_ atmosphere at 37°C. Then, the mixture was replaced by supplemented DMEM medium. Experiments were performed 48 h post-transfection (in the case of overexpression vectors) or 72 h post-transfection (in the case of shRNA vectors). For 6-well plates, 1.2 μg of DNA were used per well, whereas for 12-well plates, 0.4 μg of DNA were utilized per well.

### Lentiviral production

Lentiviruses to knockdown neuronal RTP801 were produced in HEK293 cells transfected with a pLL3.7 vector expressing shRNA against RTP801 or scrambled. Transfections were performed as aforementioned but using 100 mm plates and adding envelope and packaging plasmids (from Addgene). DNA vectors and quantities (μg/100 mm plate) necessary for lentiviral production are the following: pMD2.G (3.5), pCMV-Δr8.91 (2.5), pLL3.7-shCT (10) and pLL3.7-shRTP801 (10).

Seventy-two hours post-transfection, cell medium was collected and centrifuged for 5 min at 1500 rpm to remove cell debris. Virus‐containing medium was filtered through a 0.45 μm filter and incubated with 8.5% polyethylene glycol 6000 (Panreac AppliChem) and 0.35 M NaCl for 90 min at 4°C. The mixtures were then centrifuged at 7500 × *g* for 15 min and the pellets were resuspended in sterile 1X phosphate buffered saline (PBS) with calcium and magnesium. Lentiviruses were stored at −80°C until transduction of neurons.

### Total RNA, small RNA and tRNA isolation

WT and 5xFAD mice were euthanized by cervical dislocation and both hippocampi were dissected out and stored at −80°C until use. Total RNA was isolated using TRIzol™ Reagent (from Thermo Fisher Scientific) and following manufacturer’s instructions. Determinations of RNA quantity and quality were made with a NanoDrop™ One Spectrophotometer (from Thermo Fisher Scientific) and a 4200 TapeStation System (from Agilent Technologies), respectively. All samples showed an RNA integrity number (RIN) of 8 or higher (Supplementary Table S4). Small RNAs (sRNAs) were isolated from total RNA with the RNA Clean & Concentrator™-5 kit (from Zymo Research), according to manufacturer’s instructions. This way, we could separate sRNAs (17–200 nucleotides) from long RNAs (lRNAs, >200 nucleotides). For each sample, the sRNAs were resolved on 15% urea-polyacrylamide gels and recovered within a size window of 60–100 nucleotides, corresponding to the tRNA fraction. The lRNA and tRNA fractions were stored at −80°C until use.

### Hydrolysis-based tRNA sequencing (Hydro-tRNA-seq)

In order to determine the expression levels of precursor and mature tRNA species, a sequencing protocol called Hydro-tRNA-seq (32), specifically designed for tRNA detection and quantification, was performed at the Centre de Regulació Genòmica (CRG). Briefly, Hydro-tRNA-seq is based on the hydrolysis of the tRNAs, which generates fragments with less structure and fewer modifications that are more amenable for sequencing. In the CRG, tRNA fractions were subjected to alkaline hydrolysis, dephosphorylation and rephosphorylation with T4 Polynucleotide Kinase (PNK), to finally prepare the small RNA cDNA library as described in (32). Sequencing was performed on an Illumina HiSeq 2500 platform in a 50 base pair paired-end format (40 M reads).

The analysis of the sequencing data was performed by the Bioinformatics Unit of the CRG, using the tRNA Analysis of eXpression (tRAX) software, as described in (33).

The sequencing data underlying this article is publicly available at GEO (Gene Expression Omnibus, accession number: GSE267524 and token: mtovwwyuvzajfiv)

### Reverse transcription quantitative polymerase chain reaction (RT-qPCR)

The mRNA levels of the target genes were quantified by RT-qPCR. The NZY First-Strand cDNA Synthesis Kit (from NZYTech) was used to reverse transcribe cDNA from 1 μg of total RNA (in the case of HEK293 cells) or lRNA (in the case of WT and 5xFAD animals), according to manufacturer’s instructions. qPCR was performed with the Fast SYBR™ Green Master Mix (from Thermo Fisher Scientific) in a 7500 Real Time PCR System (from Applied Biosystems). Expression results were normalized by β-actin in human samples, and by *Hprt* in murine samples. The specific primers for qPCR are summarized in Supplementary Table S5.

### tRNA transfection

Mouse primary hippocampal neurons seeded in 12 mm coverslips were transfected at DIV 11 with the 60–100 nucleotide-sized sRNA fraction (tRNA-enriched). tRNAs were transfected using the lipid reagent Lipofectamine™ 2000 (from Thermo Fisher Scientific) according to manufacturer’s instructions. One hundred nanograms of tRNAs were added to each coverslip, as in (34). Concisely, for each well, tRNAs were mixed with 2 μl of Lipofectamine™ 2000 and 600 μl of non-supplemented Neurobasal medium and incubated for 20 min at RT. Then, the culture medium was replaced by the mixture for 4 h, and neurons were kept in a 5% CO_2_ atmosphere at 37°C. The original culture medium was stored at 4°C and was used to replace the mixture after the 4 h (it was warmed before being added to the neurons). Paraformaldehyde (PFA) fixation was performed 30 h post-transfection.

### Immunofluorescence of neuronal cultures

Mouse primary hippocampal neurons treated with tRNAs were fixed with 4% PFA (from Electron Microscopy Sciences) in PBS for 20 min at RT. Next, coverslips were permeabilized with 0.25% Triton X-100 (from Merck) in PBS for 5 min at RT and were blocked with Superblock™ Blocking Buffer for 20 min at 37°C. Primary antibodies were diluted in Superblock™ Blocking Buffer and incubated overnight at 4°C (Supplementary Table S7). The next day, coverslips were incubated with the corresponding secondary antibodies diluted in Superblock™ Blocking Buffer for 2 h at RT (Supplementary Table S7). Simultaneously, nuclei were labeled with 1:5000 bisbenzimide H-33342 trihydrochloride (Hoechst 33342, from Thermo Fisher Scientific). Between all steps coverslips were washed thrice in PBS. After secondary antibody incubation, coverslips were washed with Milli-Q water and were mounted on glass microscope slides using ProLong™ Gold Antifade Mountant (from Thermo Fisher Scientific). Samples were observed under an epifluorescent microscope (Leica AF6000, Leica Application Suite X (LAS X) software) at the Advanced optical microscopy unit (Centres Científics i Tecnològics de la UB (CCiTUB), Campus Clínic). Images were obtained with a 20× objective, and 5 images were obtained per coverslip.

### Neuron viability analysis

For the neuron viability and neuron branching analyses, the Cell Profiler software (www.cellprofiler.org, Broad Institute) (35,36) was used as previously described (37). Briefly, global nuclei and neuronal somas (MAP2^+^) were identified as independent objects, and a mask with neuronal soma was used to obtain only neuronal nuclei. ClvCas3 positive cells were identified as independent objects and were related with neuronal nuclei, with the *RelateObjects* module, to distinguish nuclei positive or negative for ClvCas3. Measures of ClvCas3 mean intensity per neuron were obtained. Nuclei classification into viable, condensed, or fragmented was based on intensity, intensity distribution, size and shape, texture, and granularity parameters, using machine learning in Cell Profiler Analyst software (38,39).

### Neuron branching analysis

The analysis of neuronal branching was performed in MAP2 images using Cell Profiler software as previously described (37). In short, the neurites of all neurons were subtracted to keep only the neuronal somas, which were identified as independent objects. Neurites were then enhanced using the enhancement method ‘line structures’ and were turned into a binary image. From this image, and using the neuronal soma as input, whole neurons were identified as independent objects. Objects at the border of the image were discarded. Neuron objects were used to mask the binary image of enhanced neurites. From the masked image, the morphological skeleton was created with the Morph module. Measurements of trunks (primary dendrites), non-trunk branches (intermediate dendrites), branch ends (terminal branches), and total tree length were obtained per neuron, using the MeasureObjectSkeleton module. Neurons from 5 fields per coverslip were analyzed.

### Statistics

All *in vitro* experiments were performed with technical replicates and were repeated three times (*n* = 3), unless otherwise stated in the figure legend. Normal distribution was assumed when all the data passed at least one of the following normality tests: D’Agostino & Pearson, Shapiro–Wilk and/or Kolmogorov–Smirnov. When two normal conditions were compared, analyses were performed using the unpaired two-tailed Student’s *t*-test (95% confidence). Welch’s correction was applied when variances were significantly different between conditions according to F-test. When Gaussian distribution was not assumed, the Mann–Whitney U test was performed. For *in vivo* experiments, in which animals are classified into four groups based on two different variables (genotype and treatment), two-way ANOVA with Bonferroni’s or Tukey’s post-hoc test was performed. Correlation analyses were measured using Pearson correlation coefficient. To detect significant outlier values, Grubbs’ and ROUT tests were used. All data are expressed as mean ± standard error of the mean (SEM). Values of *P* < 0.05 were considered statistically significant. All *P*-values are two-sided. All statistical tests were performed on GraphPad Prism 8.0, except for the receiver operating characteristic (ROC) curves and the Gene Ontology (GO) enrichment analyses, which were performed on R.

For the generation of ROC curves, protein expression values obtained from WB were used as predictor outcomes for the condition (CT or AD). R package pROC v.1.18.5 (40) was used for performance evaluation of the predictors, and ggplot2 v.3.4.4 (41) was used for graphical representation of the curves. Enrichment analysis of RTP801-interacting proteins was performed using enrichR R interface to the Enrichr database (42) and using the GO Molecular Function 2023 gene set library.

All mice bred for the experiments were used for pre-planned experiments and randomized to experimental groups. Data were collected, processed and analyzed blindly.

## Results

### RTP801 interacts with the tRNA-LC

**Figure 1.**
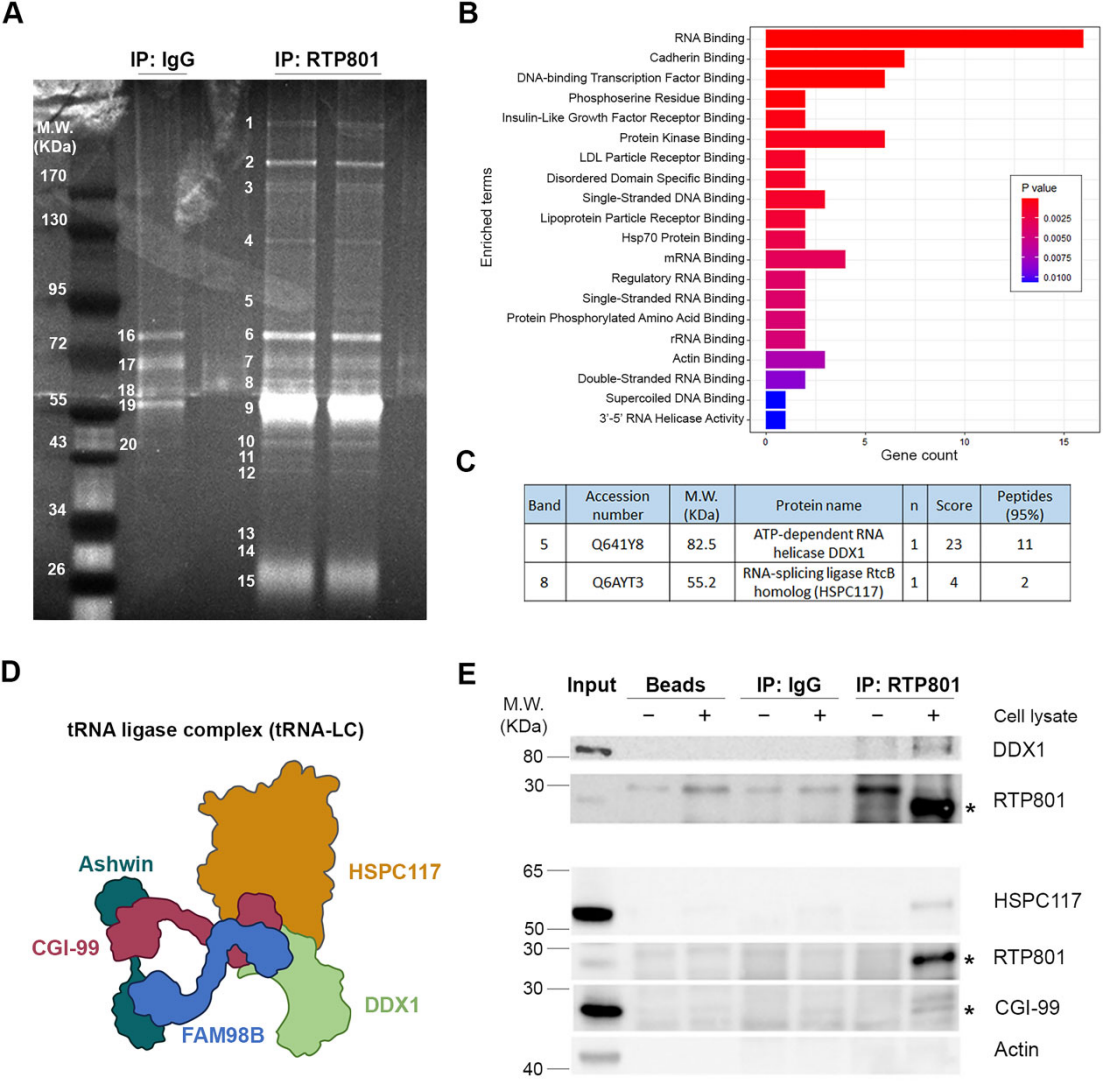
RTP801 interacts with DDX1, HSPC117 and CGI-99. (**A**) Polyacrylamide gel of RTP801 immunocomplexes. RTP801 was immunoprecipitated from rat cortical neuronal cultures (DIV 14) and the resulting immunocomplexes were resolved in a SDS-PAGE gel. The bands were stained, numbered and cut for the subsequent analysis by MS. (**B**) GO biological process enrichment analysis of RTP801 interactors. For each enriched category, the Fisher’s exact test *P*-value is calculated, and bars are filled according to it. The top 20 enriched GO molecular function terms are plotted. (**C**) Table showing the accession number (Uniprot database), molecular weight (M.W.), number of times identified (*n*), score and the number of peptides detected for DDX1 and HSPC117 in the MS analysis. (**D**) Schematic representation of the tRNA-LC components. (**E**) Immunoprecipitation of RTP801 in HEK293 cells. After a 2-h treatment with DSP, HEK293 cells were harvested and endogenous RTP801 was immunoprecipitated. Some lysates were incubated with beads or beads bound to normal IgGs as negative controls. Samples were then analyzed by WB. The asterisks indicate the bands corresponding to RTP801 (≈ 28 kDa) and CGI-99 (≈27 kDa); *n* = 2.

To investigate potential novel functions of RTP801, we studied its interactors by MS. Briefly, RTP801 was immunoprecipitated from rat primary cortical neuronal cultures (DIV14) and the resulting immunocomplexes were resolved in an SDS-PAGE gel. The bands were numbered (Figure 1A), cut and analyzed by MS. We then performed a GO biological process enrichment analysis of the 43 obtained RTP801 interactors and found that 16 of them were RNA-binding proteins (Figure 1B). Interestingly, the bands ‘5’ and ‘8’ corresponded to DDX1 and HSPC117, respectively (Figure 1C). These two proteins are part of the tRNA-LC (Figure 1D) first described by Popow *et al.* in 2011 (18), which ligates the tRNA exons generated during tRNA splicing. To validate the MS results, endogenous RTP801 was immunoprecipitated in HEK293 cells and both DDX1 and HSPC117 were detected by WB. Moreover, CGI-99, another member of this complex, was also pulled down. On the contrary, actin, which was included as negative control, was not detected (Figure 1E).

### RTP801 does not affect the protein levels of the tRNA-LC effectors

**Figure 2.**
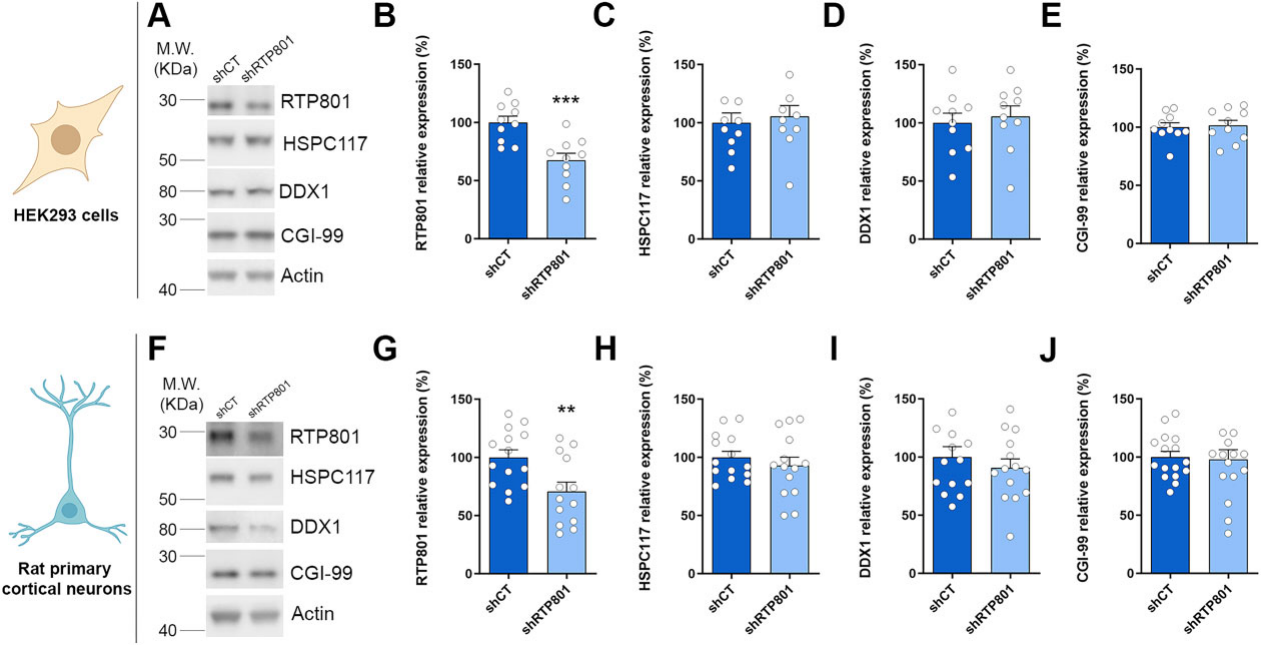
RTP801 does not affect the protein levels of the tRNA-LC effectors. (**A**) WB for RTP801, HSPC117, DDX1, CGI-99, and actin as loading control in HEK293 cells transfected with shRNA against RTP801. (**B–E**) Densitometric quantification of RTP801 (B), HSPC117 (C), DDX1 (D) and CGI-99 (E) results as in (A) (RTP801: *t*_18_ = 4.064, *P* = 0.0007; HSPC117: *t*_18_ = 0.432, *P* = 0.6693; DDX1: *t*_18_ = 0.4452, *P* = 0.6615; CGI-99: *t*_18_ = 0.2417, *P* = 0.8118). (**F**) WB for RTP801, HSPC117, DDX1, CGI-99, and actin as loading control in rat cortical neurons infected with shRNA against RTP801-containing lentiviruses. (**G–J**) Densitometric quantification of RTP801 (G), HSPC117 (H), DDX1 (I) and CGI-99 (J) results as in (F) (RTP801: *t*_25_ = 2.852, *P* = 0.0086; HSPC117: *t*_26_ = 0.7986, *P* = 0.4317; DDX1: *t*_26_ = 0.7879, *P* = 0.4379; CGI-99: *t*_30_ = 0.2107, *P* = 0.8346). All data are analyzed with the unpaired two-tailed Student’s *t*-test, and all data are represented as mean ± SEM. Values represent technical replicates of three independent experiments. Values were excluded when they were classified as outliers with either the ROUT or the Grubbs’ test from Graphpad Prism software. **P < 0.01 and ***P < 0.001.

To investigate whether RTP801 could affect the protein stability of the subunits of the complex, RTP801 was downregulated about a 30% using short hairpin RNA (shRNA) in both HEK293 cells (Figure 2A–E) and rat primary cortical neurons (Figure 2F–J). By WB, we observed that RTP801 knockdown does not alter the protein levels of HSPC117, DDX1 nor CGI-99. Therefore, these results discard an eventual proteostatic regulation of RTP801 over these complex members.

### RTP801 inhibits the splicing of *XBP1* mRNA *in vitro*

To explore whether the interaction of RTP801 with the tRNA-LC could impair its mRNA ligase activity, we monitored the unconventional splicing of *XBP1* mRNA. *XBP1* splicing occurs when unfolded or misfolded proteins accumulate in the ER and the UPR is triggered. *XBP1* splicing can be assessed by studying the ratio between the mRNA levels of the spliced (*XBP1s*) and the unspliced (*XBP1u*) forms.

We first investigated whether HSPC117 knockdown with a shRNA impaired *XBP1* mRNA splicing in HEK293 cells. As previously described (43), HSPC117 silencing alone was not sufficient to alter the ratio between *XBP1s* and *XBP1u* mRNA (Supplementary Figure S1A–D), which suggests that low levels of HSPC117 are sufficient to mediate an efficient ligation of *XBP1* exons.

**Figure 3.**
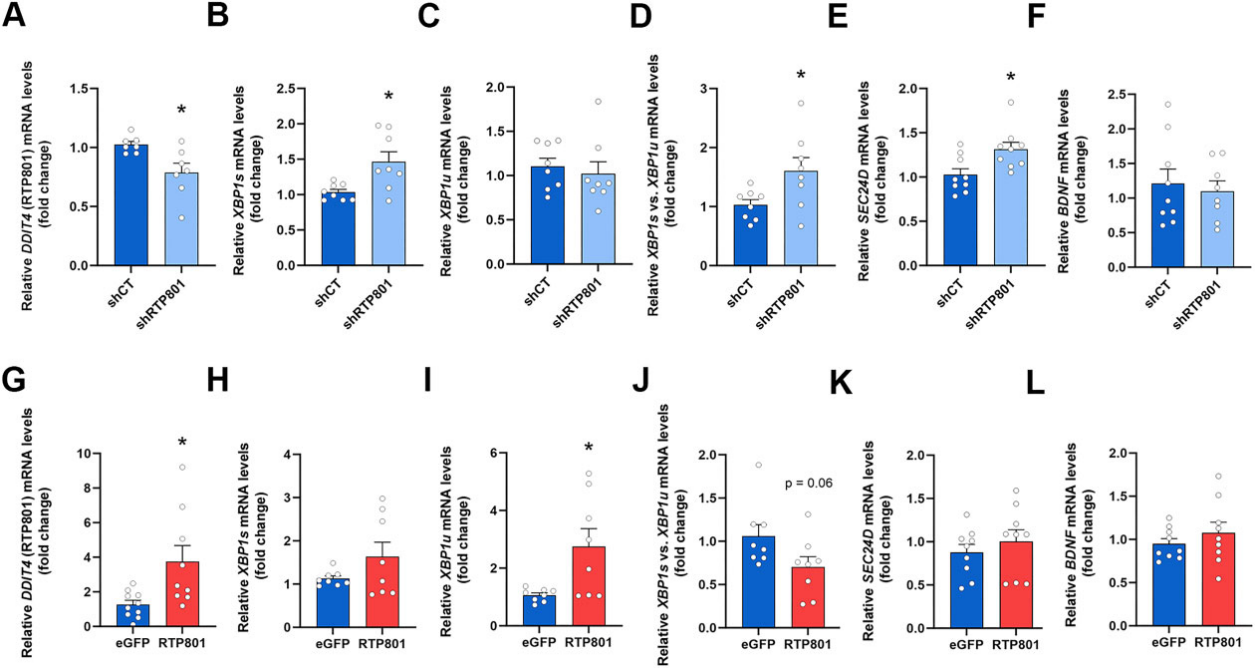
RTP801 inhibits the splicing of *XBP1 in vitro*. HEK293 cells were transfected with shCT, shRTP801, eGFP or eGFP-RTP801. Two or three days later, RNA was extracted, retrotranscribed and RT-qPCR was performed. (**A–F**) RT-qPCR results in HEK293 cells with downregulation of RTP801. Relative expression of *DDIT4* (RTP801 coding gene) (A), *XBP1s* (B), *XBP1u* (C), *XBP1s/XBP1u* (D), *SEC24D* (E) and *BDNF* (F) (*DDIT4*: *t*_7.326_ = 2.809, *P* = 0.0250; *XBP1s*: *t*_8.129_ = 2.936, *P* = 0.0185; *XBP1u*: *t*_14_ = 0.5131, *P* = 0.6159; *XBP1s/XBP1u*: *t*_9.129_ = 2.370, *P* = 0.0416; *SEC24D*: *t*_16_ = 2.757, *P* = 0.0140; *BDNF*: *t*_15_ = 0.4246, *P* = 0.6772). (**G–L**) RT-qPCR results in HEK293 cells with upregulation of RTP801. Relative expression of *DDIT4* (RTP801 coding gene) (G), *XBP1s* (H), *XBP1u* (I), *XBP1s/XBP1u* (J), *SEC24D* (K) and *BDNF* (L) (*DDIT4*: *t*_9.087_ = 2.618, *P* = 0.0277; *XBP1s*: *t*_7.478_ = 1.520, *P* = 0.1697; *XBP1u*: *t*_7.228_ = 2.714, *P* = 0.0291; *XBP1s/XBP1u*: *t*_14_ = 2.003, *P* = 0.0649; *SEC24D*: *t*_16_ = 0.7845, *P* = 0.4442; *BDNF*: *t*_16_ = 0.9284, *P* = 0.3670). *ACTB* (β-actin) was used to normalize the expression of all genes. All data are analyzed with the unpaired two-tailed *t*-test. Welch’s correction was applied in panels (A, B, D, G, H, and I) because variances were unequal between the two conditions. All data are represented as mean ± SEM. Values represent technical replicates of three independent experiments. Values were excluded when they were classified as outliers with either the ROUT or the Grubbs’ test from Graphpad Prism software; *P < 0.05.

However, knocking down RTP801 by a 25% in HEK293 cells (Figure 3A) caused a significant increase in *XBP1s* mRNA (Figure 3B) without altering the levels of *XBP1u* mRNA (Figure 3C), leading to an elevation of the *XBP1s*/*XBP1u* ratio (Figure 3D). Interestingly, we also observed an increase in *SEC24D* mRNA, a target gene of XBP1s as a transcription factor (44,45) (Figure 3E), but we found no changes in another target gene, such as *BDNF* (46) (Figure 3F). On the contrary, RTP801 overexpression (Figure 3G) resulted in a significant accumulation of *XBP1u* mRNA with no changes in *XBP1s*, mRNA which resulted in a reduced *XBP1s/XBP1u* ratio (Figure 3H–J). Regarding *SEC24D* and *BDNF* mRNA, no differences were observed when RTP801 was upregulated (Figure 3K–L). These results suggest that RTP801 inhibits the unconventional splicing of *XBP1*, since its levels inversely correlate with the *XBP1s/XBP1u* ratio.

### XBP1 splicing is impaired in the hippocampus of AD patients

**Figure 4.**
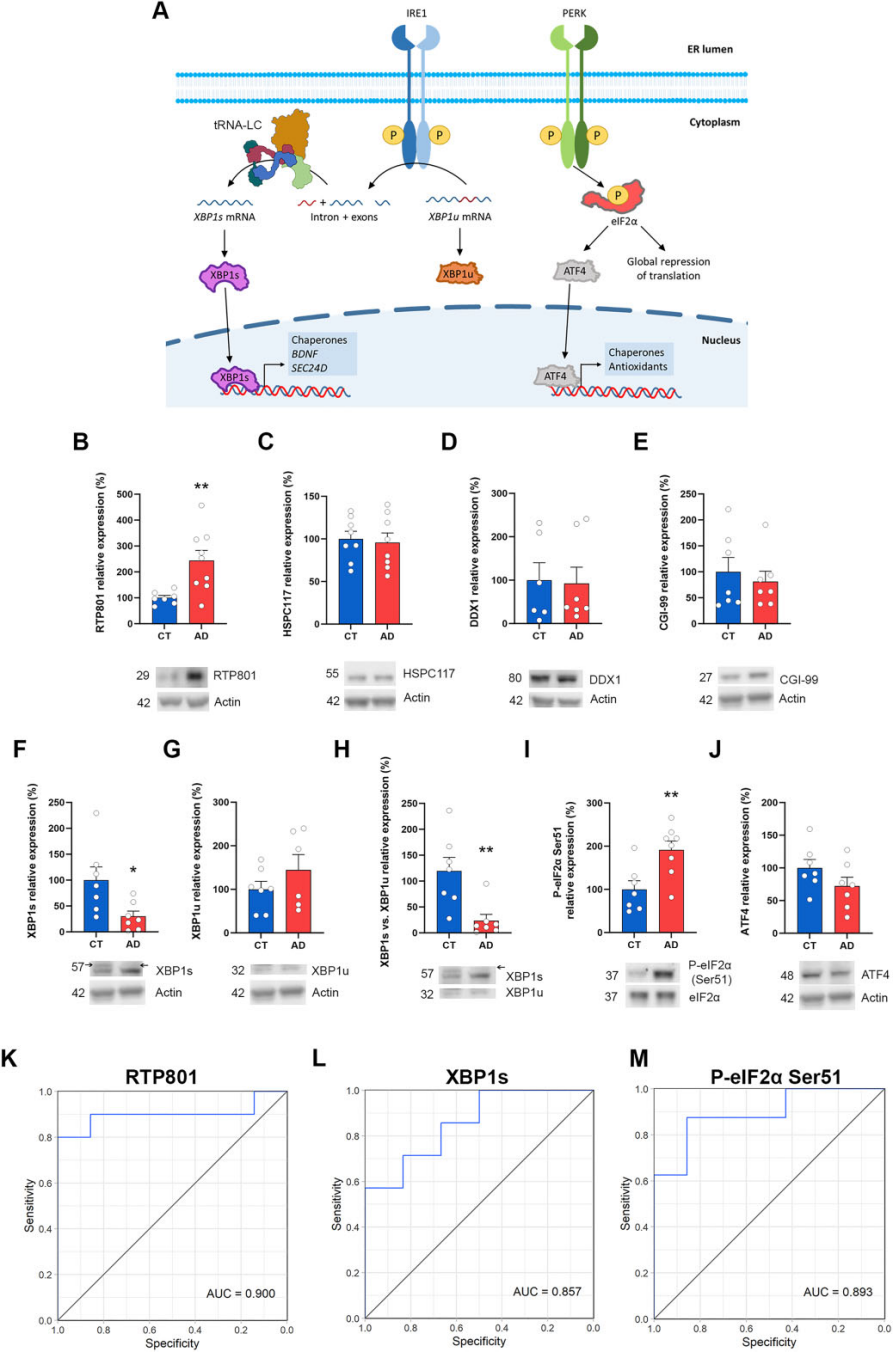
Reduced XBP1 splicing in the hippocampus of AD patients. (**A**) Graphical representation of the IRE1 and the PERK branches of the UPR. Accumulation of unfolded or misfolded proteins leads to the activation of the UPR. The effector of the IRE1 branch is XBP1s, a transcription factor whose mRNA requires the ligation by the tRNA-LC to be translated. The effector of the PERK branch is phosphorylated eIF2α, which promotes the expression of ATF4. (**B–J**) WB and densitometric quantification for RTP801 (B), HSPC117 (C), DDX1 (D), CGI-99 (E), XBP1s (F), XBP1u (G), XBP1s/XBP1u (H), p-eIF2α Ser51 (I), ATF4 (J) and actin as loading control in human postmortem hippocampal samples. (RTP801: *t*_8.856_ = 3.529, *P* = 0.0066; HSPC117: *t*_14_ = 0.2921, *P* = 0.7745; DDX1: U = 18, *P* = 0.7308; CGI-99: *t*_12_ = 0.5519, *P* = 0.5912; XBP1s: *t*_7.855_ = 2.556, *P* = 0.0344; XBP1u: *t*_11_ = 1.168, *P* = 0.2675; XBP1s/XBP1u: U = 3, *P* = 0.0041; p-eIF2α Ser51: *t*_13_ = 3.183, *P* = 0.0072; ATF4: *t*_12_ = 1.485, *P* = 0.1634). All data are analyzed with the unpaired two-tailed *t*-test (except for panel [D and H]). The arrows in panels (F and H) indicate the specific band for XBP1s (≈ 57 kDa). Welch’s correction was applied in panels (B and F) because variances were unequal between the two conditions. Data in panels (D and H) were analyzed with Mann–Whitney U test because values did not pass the normality test. All data are represented as mean ± SEM. Values were excluded when they were classified as outliers with either the ROUT or the Grubbs’ test from GraphPad Prism software. *P < 0.05 and **P < 0.01. (**K–M**) ROC curves of RTP801 (K), XBP1s (L) and p-eIF2α Ser51 (M) obtained from the protein expression values from the densitometric analyses in (B), (F) and (I), respectively.

Next, we investigated whether the inhibitory effect of RTP801 upon XBP1 splicing occurred in the human brain and whether it was impaired in AD. Interestingly, although little is known about XBP1s in the human disease, XBP1s has been recently described to prevent synaptic dysfunction and memory loss in different mouse models of AD, including the 5xFAD (47,48). Thus, we explored the splicing of XBP1 as well as the PERK branch of the UPR (both represented in Figure 4A) in hippocampal postmortem samples from AD patients and age-matched controls (CT).

By WB, we confirmed the elevation of RTP801 in hippocampal AD samples, as we previously described (8) and we found no significant changes in HSPC117, DDX1 or CGI-99 protein levels (Figure 4B–E). Next, we assessed the levels of XBP1s and XBP1u by WB because human brain postmortem RNA samples showed low RNA integrity number (RIN values), around 6, and far from the acceptable RIN of 8. Nonetheless, we found a drastic reduction in the protein levels of XBP1s with no changes in the levels of XBP1u, leading to a decreased XBP1s/XBP1u ratio in AD patients (Figure 4F–H). Regarding the PERK branch of the UPR, we found significantly increased phosphorylation of eIF2α on serine 51 in AD patients, which was not accompanied by changes in the levels of the transcription factor ATF4 (Figure 4I–J). These results suggest that, while the PERK branch of the UPR is active in AD patients, the IRE1 branch, and specifically, XBP1 splicing seems to be more affected. Interestingly, the protein levels of RTP801, XBP1s and P-eIF2α Ser51 were very good classifiers of the presence or the absence of the disease, as determined by the area under the curve (AUC), being RTP801 the best marker (Figure 4K–M).

### Genetic normalization of hippocampal RTP801 levels in the 5xFAD mouse model of AD promotes the splicing of *XBP1* and the transcription of *BDNF*

**Figure 5.**
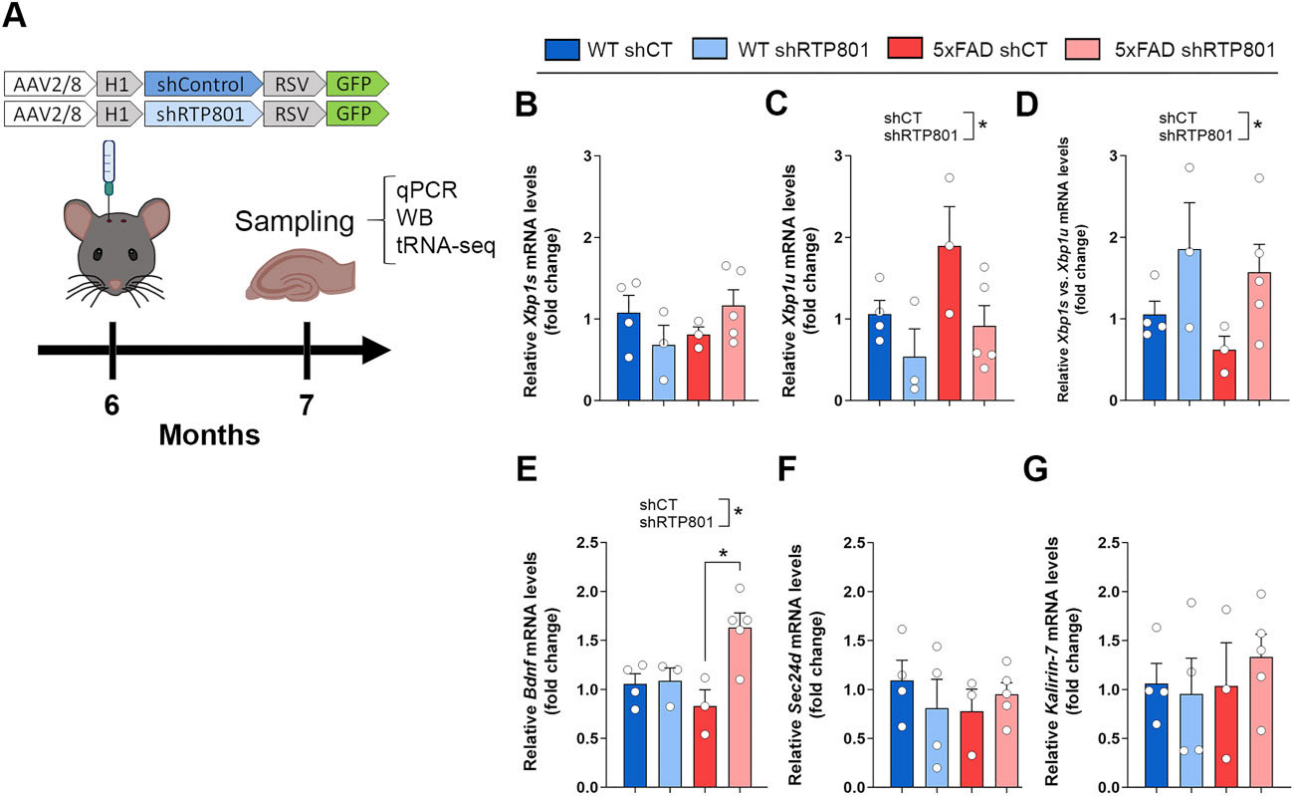
RTP801 downregulation in hippocampal neurons promotes *Xbp1* splicing in the 5xFAD mouse model of AD. (**A**) Timeline of mice surgery and sampling. Six-month-old WT and 5xFAD mice were bilaterally injected in the dorsal hippocampus with neuron-directed AAVs containing shCT or shRTP801 and GFP. Four weeks later animals were euthanized, and the hippocampi were obtained for RT-qPCR, WB and sequencing. (**B–G**) RT-qPCR results relativized to *Hprt*. Relative expression of *Xbp1s* (B), *Xbp1u* (C), *Xbp1s/Xbp1u* (D), *Bdnf* (E), *Sec24d* (F) and *Kalirin-7* (G) (*Xbp1u*: treatment effect: *F*_(1, 11)_ = 6.109, *P* = 0.0310; *Xbp1s/XBP1u:* treatment effect: *F*_(1, 11)_ = 6.375, *P* = 0.0282; *Bdnf:* treatment effect: *F*_(1, 11)_ = 7.936, *P* = 0.0168, interaction effect: *F*_(1, 11)_ = 6799, *P* = 0.0244). Data are means ± SEM. Each value represents one animal. In all panels two-way ANOVA with Tukey’s post-hoc test was performed. Values were excluded when they were classified as outliers with either the ROUT or the Grubbs’ test from Graphpad Prism software. *P < 0.05.

Next, we investigated whether RTP801 protein levels could directly affect *Xbp1* mRNA unconventional splicing in the 5xFAD mouse model of AD. In previous results, we found that RTP801 was increased in hippocampal synaptosomes of 5xFAD mice, but not in total lysates, and we reported that genetic silencing of RTP801 in hippocampal neurons prevented cognitive deterioration and neuroinflammation (8). Hence, 6-month-old wild-type (WT) and 5xFAD mice were stereotactically injected in the dorsal hippocampus with neuron-targeting AAVs expressing scrambled shRNA (shCT) or shRNA against RTP801 (shRTP801) as in (8). This approach generated four groups of mice, namely, WT shCT, WT shRTP801, 5xFAD shCT and 5xFAD shRTP801. One month after injection, mice were euthanized and the hippocampi were collected to perform RT-qPCR, WB and Hydro-tRNA-seq (depicted in Figure 5A).

In our previous work, we reported that shRTP801-containing AAVs promote a 20–30% reduction in the levels of RTP801 in these animals (8). In this line, we found a similar knockdown when assessed by WB and RT-qPCR (Supplementary Figure S2A–C). Regarding *Xbp1* splicing, by RT-qPCR we found that the levels of *Xbp1s* mRNA are not altered in 5xFAD mice compared to WT animals (Figure 5B). However, *Xbp1u* mRNA tends to accumulate in 5xFAD mice, and, interestingly, RTP801 silencing significantly reduces the levels of the unspliced form of *Xbp1* (Figure 5C). As for the ratio between *Xbp1s* and *Xbp1u*, as a readout of splicing efficiency, we observed that silencing RTP801 promotes *Xbp1* mRNA splicing (Figure 5D), in line with our *in vitro* results (see Figure 3D). In addition, when we monitored the expression of XBP1s transcriptional targets, we observed that RTP801 downregulation in 5xFAD mice increased the levels of *Bdnf mRNA*, which plays an essential role in neuronal survival and growth, as well as in learning and memory (49) (Figure 5E). On the other hand, the mRNA levels of other XBP1s targets, such as *Sec24d* (44,45) (Figure 5F) and *Kalirin-7* (48) (Figure 5G) were invariable between experimental groups. Altogether, these results suggest that RTP801 impairs the activity of the tRNA-LC over *Xbp1* splicing in WT and 5xFAD mice. We conclude that RTP801 silencing in the 5xFAD hippocampal neurons is contributing to *Bdnf* mRNA elevation, but this does not exclude other contributing mechanisms to this phenomenon, such as the transcellular cross-talk between neurons and BDNF-producing astrocytes (50).

### RTP801 prevents the accumulation of intron-containing pre-tRNAs in 5xFAD mice

To investigate whether RTP801 function over the tRNA-LC was specific for *Xbp1* mRNA or whether it could be extended to intron-containing RNAs, RNA from WT and 5xFAD mice with or without neuronal silencing of RTP801 was isolated to perform Hydro-tRNA-seq (see Figure 5A for timeline). Briefly, 60 to 100 nucleotide-sized RNA was isolated and hydrolyzed for cDNA library preparation and sequencing. This fraction encompasses both pre-tRNAs and mature tRNAs but contains tiny amounts of other small RNAs such as ribosomal RNAs (rRNAs) or small nucleolar RNAs (snoRNAs) (51). We started by confirming the correct sequencing of samples by characterizing the obtained reads (Supplementary Figure S3). The amount (>20 million) and proportion (>60%) of mappable reads were satisfactory (Supplementary Figure S3A), and we found that most of our merged reads mapped mature (51.8%) or precursor tRNAs (11.6%) (Supplementary Figure S3B), in consonance with bibliography (32). Interestingly, mitochondrial tRNAs (mt-tRNAs) represented the 15.3% of the reads. Other small RNAs were also present but in a much lesser extent, as anticipated. No significant differences in the proportion of RNA types were found when animals were compared, either individually or grouped per condition.

**Figure 6.**
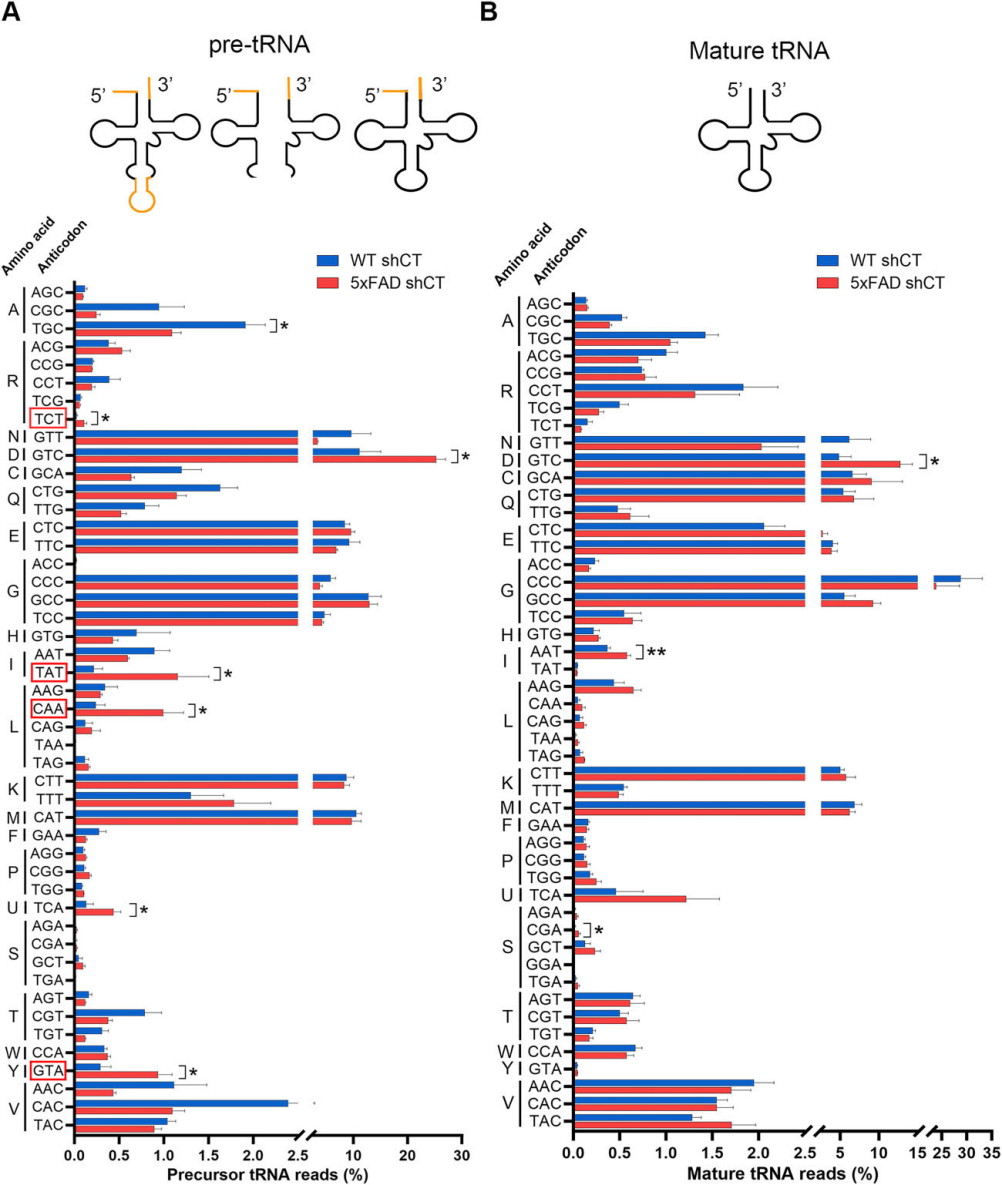
5xFAD mice accumulate intron-containing pre-tRNAs. tRNAs were isolated from 7-month-old WT and 5xFAD mice and sequenced by Hydro-tRNA-seq. The percentage of normalized counts for precursor (**A**) and mature (**B**) tRNA is depicted, classified by amino acid and anticodon (pre-tRNA-Ala-TGC: *t*_6_ = 2.692, *P* = 0.0360; pre-tRNA-Arg-TCT: *t*_5_ = 3.364, *P* = 0.0200; pre-tRNA-Asp-GTC: *t*_6_ = 2.635, *P* = 0.0388; pre-tRNA-Ile-TAT: *t*_5_ = 2.986, *P* = 0.0306; pre-tRNA-Leu-CAA: *t*_6_ = 3.440, *P* = 0.0138; pre-tRNA-Sec-TCA: *t*_6_ = 2.496, *P* = 0.0468; pre-tRNA-Tyr-GTA: *t*_6_ = 3.264, *P* = 0.0172) (tRNA-Asp-GTC: *t*_6_ = 3.332, *P* = 0.0158; tRNA-Ile-AAT: *t*_6_ = 3.814, *P* = 0.0088; tRNA-Ser-CGA: *t*_6_ = 2.771, *P* = 0.0324). The red rectangles surrounding certain anticodons indicate that at least one of those pre-tRNA isodecoders has an intron. Data are means ± SEM. In all comparisons Student’s *t*-test was performed. Values were excluded when they were classified as outliers with either the ROUT or the Grubbs’ test from Graphpad Prism software. *P < 0.05 and **P < 0.01.

After confirming the favorable outcome of the sequencing, we compared the pool of precursor and mature tRNAs between WT shCT and 5xFAD shCT mice, classifying the tRNA species by their anticodon (Figure 6A,B). Strikingly, all pre-tRNA isodecoders with intron-containing species (R/arginine-TCT, I/isoleucine-TAT, L/leucine-CAA, and Y/tyrosine-GTA) were significantly accumulated in 5xFAD mice (Figure 6A, indicated with a red square in the Y axis), suggesting an impairment in tRNA splicing in 5xFAD mice. This accumulation, however, was not observed as for the mature tRNAs. Significant differences were also found in the levels of precursor tRNA-Ala-TGC, tRNA-Asp-GTC and tRNA-Sec-TCA, as well as in mature tRNA-Asp-GTC, tRNA-Ile-AAT and tRNA-Ser-CGA (Figure 6B). Interestingly, no differences were detected when we compared the abundance of the mt-tRNA species between WT shCT and 5xFAD shCT mice (Supplementary Figure S4), suggesting that the alteration in the pool of tRNAs in 5xFAD mice might be limited to a very specific and well-defined subpopulation of tRNAs, namely those with intron.

**Figure 7.**
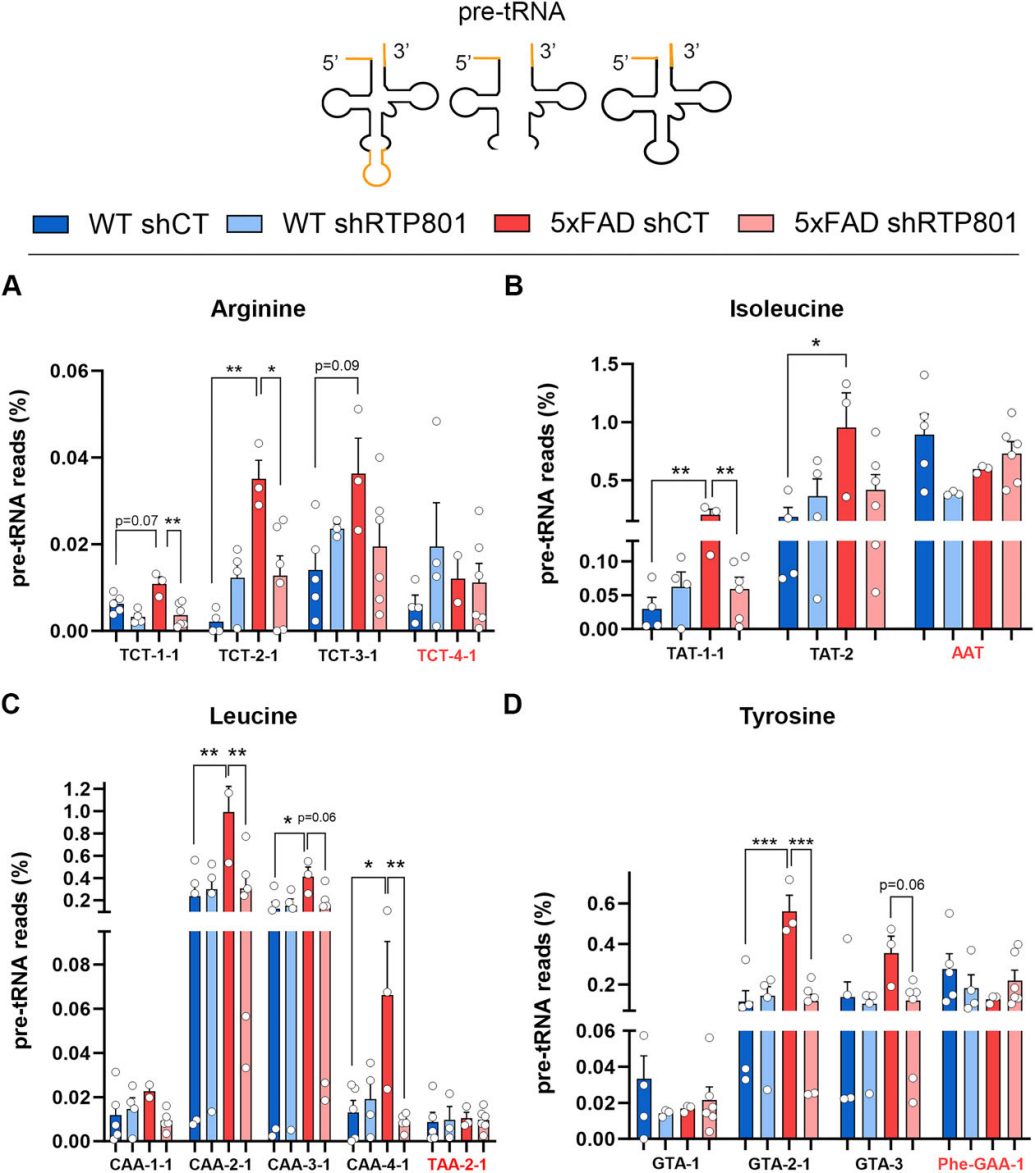
RTP801 downregulation in hippocampal neurons prevents accumulation of intron-containing pre-tRNAs in 5xFAD mice. The percentage of normalized counts for pre-tRNA is depicted, classified by amino acid and anticodon. Different pre-tRNA species within an isodecoder family are represented. Pre-tRNA species in red do not have an intron and are included as a control. Since all tyrosine-accepting pre-tRNAs have intron, pre-tRNA-Phe-GAA-1 (also accepts an aromatic amino acid) was included as a control. Relative expression of pre-tRNA-Arg-TCT (**A**), pre-tRNA-Ile-TAT (**B**), pre-tRNA-Leu-CAA (**C**), and pre-tRNA-Tyr-GTA (**D**) (pre-tRNA-Arg-TCT-1–1: genotype effect: F_(1, 14)_ = 4.942, *P* = 0.0432, treatment effect: F_(1, 14)_ = 20.64, *P* = 0.0005; pre-tRNA-Arg-TCT-2–1: genotype effect: F_(1, 13)_ = 15.37, *P* = 0.0018, interaction effect: F_(1, 13)_ = 14.52, *P* = 0.0022; pre-tRNA-Arg-TCT-3–1: interaction effect: F_(1, 13)_ = 4.876, *P* = 0.0458; pre-tRNA-Ile-TAT-1–1: genotype effect: F_(1, 13)_ = 11.69, *P* = 0.0046, treatment effect: F_(1, 13)_ = 5.093, *P* = 0.0419, interaction effect: F_(1, 13)_ = 12.52, *P* = 0.0036; tRNA-Ile-TAT-2: genotype effect: F_(1, 13)_ = 6.102, *P* = 0.0281; pre-tRNA-Leu-CAA-2–1: genotype effect: F_(1, 14)_ = 10.75, *P* = 0.0055, treatment effect: F_(1, 14)_ = 7.621, *P* = 0.0153, interaction effect: F_(1, 14)_ = 10.46, *P* = 0.0060; pre-tRNA-Leu-CAA-3–1: genotype effect: F_(1, 14)_ = 5.081, *P* = 0.0407, interaction effect: F_(1, 14)_ = 4.977, *P* = 0.0426; pre-tRNA-Leu-CAA-4–1: genotype effect: F_(1, 13)_ = 4.953, *P* = 0.0444, treatment effect: F_(1, 13)_ = 7.206, *P* = 0.0187, interaction effect: F_(1, 13)_ = 11.05, *P* = 0.0055; pre-tRNA-Tyr-GTA-2–1: genotype effect: F_(1, 14)_ = 17.27, *P* = 0.0010, treatment effect: F_(1, 14)_ = 17.11, *P* = 0.0010, interaction effect: F_(1, 14)_ = 22.18, *P* = 0.0003; pre-tRNA-Tyr-GTA-3: treatment effect: F_(1, 14)_ = 5.218, *P* = 0.0385). Data are means ± SEM. In all comparisons two-way ANOVA with Tukey’s post hoc test was performed. Each value represents one animal. Values were excluded when they were classified as outliers with either the ROUT or the Grubbs’ test from Graphpad Prism software; *P < 0.05, **P < 0.01, and ***P < 0.001.

To investigate whether the levels of RTP801 could influence tRNA splicing *in vivo*, within the four isodecoder families that have pre-tRNAs with intron, we compared the proportion of reads that mapped pre-tRNAs between all our experimental groups (Figure 7A–D). We also assessed the abundance of other pre-tRNA species without intron (either isodecoders, isoacceptors, or related species) as negative controls. Additional information about the murine pre-tRNAs that contain intron can be found in Supplementary Table S6. As observed in Figure 6A, virtually all intron-containing pre-tRNAs accumulate in 5xFAD mice. Remarkably, this accumulation is most of the times prevented when RTP801 is silenced in hippocampal neurons. For instance, both precursor tRNA-Arg-TCT-2–1 and tRNA-Leu-CAA-2–1 are significantly increased in 5xFAD animals and RTP801 downregulation prevents this aberrant accumulation. Notably, this phenotype is not found in those pre-tRNAs that do not contain intron. When we studied the levels of the mature forms of these tRNAs, we found no significant differences between conditions (Supplementary Figure S5A–D). These results indicate that knocking down RTP801 specifically in hippocampal neurons affects tRNA splicing and therefore modulates the pool of pre-tRNA in this brain region without altering the pool of mature tRNAs.

### The tRNA-enriched sRNA fraction from 5xFAD mice increases dendrite branching in hippocampal cultured neurons

**Figure 8.**
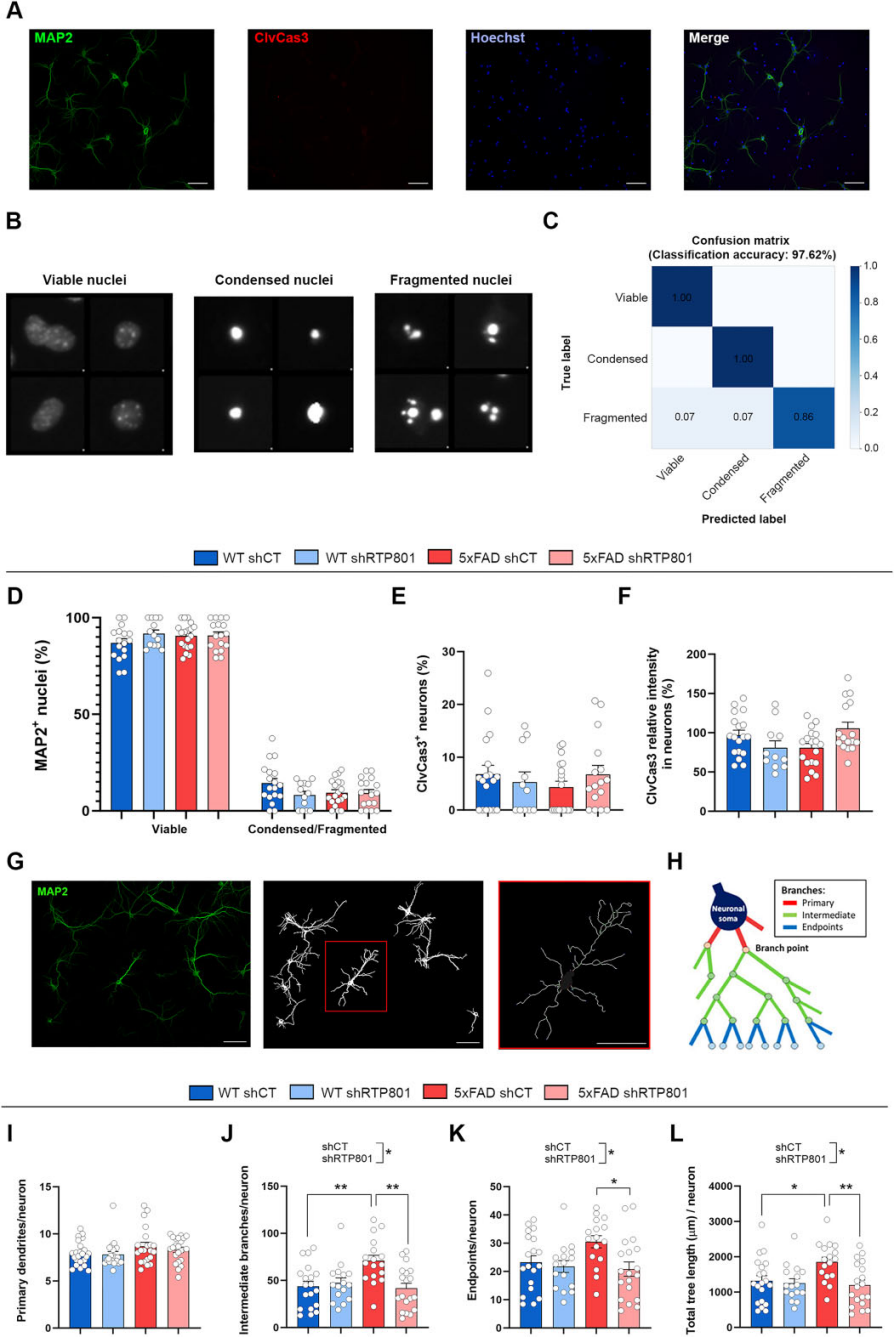
The tRNA-enriched sRNA fraction from 5xFAD mice increases dendrite branching in hippocampal cultured neurons without affecting neuron viability. (**A**) Representative images of tRNA-transfected mouse hippocampal neurons stained with MAP2, ClvCas3, and Hoechst 33342. (**B**) Classification of neuronal nuclei into viable, condensed or fragmented with Cell Profiler Analyst. (**C**) Confusion matrix for the nuclei classification in (B). (**D**) Proportion of viable and condensed/fragmented neuronal nuclei. (**E**) Percentage of ClvCas3^+^ neurons. (**F**) ClvCas3 mean intensity in neurons. (**G**) From MAP2 images, neurons not touching the borders of the image were identified as independent objects, and the neuron skeleton was obtained. (**H**) Schematic representation of the different types of branches found in a neuron (adapted from (37)). (**I–K**) Average number of primary dendrites (I), intermediate branches (J) and endpoints (K) per neuron (Intermediate branches: treatment effect: *F*_(1, 67)_ = 5.610, *P* = 0.0207, interaction effect: *F*_(1, 67)_ = 9.263, *P* = 0.0033; endpoints: treatment effect: *F*_(1, 66)_ = 5.863, *P* = 0.0182). (**L**) Average total tree length (μm) per neuron (treatment effect: F_(1, 68)_ = 6.767, *P* = 0.0114, interaction effect: *F*_(1, 68)_ = 4.532, *P* = 0.0369); Scale bar = 50 μm. Data are means ± SEM. In all comparisons two-way ANOVA with Tukey's *post hoc* test was performed. Each value represents the mean of one microscope image, obtained from two independent experiments with technical replicates. Values were excluded when they were classified as outliers with either the ROUT or the Grubbs’ test from Graphpad Prism software; *P < 0.05 and **P < 0.01.

Finally, we studied whether the 60–100 nucleotide-sized, tRNA-enriched sRNA from our mice was toxic for mouse hippocampal neurons or had any effect on dendrite branching. Thus, cultured neurons were transfected with 100 ng of sRNA (see Supplementary Figure S4 for sRNA composition) for 30 h. Then, cultures were fixed and immunostained with antibodies against MAP2 and cleaved caspase 3 (ClvCas3), and nuclei were stained with Hoechst 33342 (Figure 8A). Neuronal nuclei were classified into viable, condensed or fragmented (Figure 8B) using a machine learning pipeline that showed an accuracy of 97.62% (Figure 8C). We found that any of the tRNA-enriched sRNAs affected neuron survival, assessed by the proportion of viable nuclei (Figure 8D). Moreover, the proportion of ClvCas3 + neurons was stable between conditions (Figure 8E), as well as the mean intensity of ClvCas3 immunostaining in neurons (Figure 8F). We next evaluated neuron arborization by obtaining the neuron skeleton (Figure 8G) and quantifying the primary dendrites, intermediate branches, endpoints and the total tree length (Figure 8H). Strikingly, we observed that the tRNA-enriched fraction derived from 5xFAD shCT mice increased the number of intermediate branches and endpoints in cultured neurons, which led to an augmented tree length (Figure 8I–L). These results suggest that the pre-tRNAs and mature tRNAs from 5xFAD mice have an impact on neuron branching, which is prevented by RTP801 silencing in the source neurons.

## Discussion

Here, we found that the stress-induced protein RTP801 interacts with the tRNA-LC, specifically with HSPC117, DDX1 and CGI-99, but it does not regulate their protein levels, neither in rat cortical neurons nor in human HEK293 cells. Interestingly, RTP801 downregulation in HEK293 cells promoted the splicing of *XBP1* mRNA, while RTP801 overexpression inhibited it. Similarly, we found that XBP1 splicing is impaired in the hippocampus of AD patients, where RTP801 levels are elevated. Remarkably, *Xbp1* splicing is also modulated by RTP801 in the 5xFAD hippocampus, as well as the expression of *Bdnf* mRNA. In these samples, we also detected an accumulation of intron-containing pre-tRNAs, which does not correlate with an accumulation of mature tRNAs, suggesting an impairment in tRNA splicing in 5xFAD mice. Indeed, in most cases RTP801 downregulation prevented the accumulation of pre-tRNAs with intron. We also found that the tRNA-enriched sRNA fraction derived from the hippocampus of 5xFAD shCT mice induced increased arborization in hippocampal cultured neurons, but RTP801 silencing in the source neurons prevented it.

In this work, we observed that RTP801 interacts mainly with RNA-binding proteins, such as DDX1 and HSPC117, two members of the tRNA-LC. We confirmed this interaction both in rat primary cortical neurons and in HEK293 cells, by two different approaches. Interestingly, in HEK293 cells, we also found that RTP801 interacts with CGI-99, another member of the tRNA splicing complex. This complex was first described by Popow *et al.* in 2011 (18) as a pentameric complex formed by HSPC117, DDX1, CGI-99, ASW and FAM98B. However, in recent years new regulators of the activity of the complex (both competitors and cofactors) have been described, such as archease (52,53), PYROXD1 (54) or CNP (55), among others (reviewed in (56)). The fact that these proteins were not detected in the co-immunoprecipitation experiments of Popow *et al.* (18) suggests that they form weak or transient interactions with the complex. In line with this, we hypothesize that RTP801 is an elusive interactor of the tRNA-LC, mostly due to its short half-life (2–5 min) (57,58). Indeed, this is corroborated by the fact that we only were able to detect RTP801 interacting with the complex when we treated our cell cultures with DSP, a chemical cross-linker.

We found that RTP801 does not modulate the protein levels of HSPC117, DDX1 or CGI-99, neither in HEK293 cells nor in rat primary cortical neurons, which indicates that RTP801 does not seem to regulate the translation or the degradation of such proteins. However, whether RTP801 impairs the assembly and hence, the stability of the tRNA-LC remains unknown.

The unconventional splicing of *XBP1* is a key event during the UPR, since XBP1s target genes promote ER homeostasis restoration (28). Jurkin *et al.* (19) found that the tRNA-LC, and specifically HSPC117, is responsible for the ligation of *XBP1* mRNA exons, thus generating *XBP1s*. However, in their work, the knockdown of HSPC117 alone was not sufficient to impair *XBP1* splicing, as previously described (43). In the same line, we found that HSPC117 knockdown in HEK293 cells did not compromise *XBP1* splicing. Only when HSPC117 and its cofactor archease were simultaneously depleted, a significant decrease in *XBP1* splicing was observed (19). Nonetheless, in this work we demonstrated that the knockdown of RTP801 alone promoted *XBP1* splicing in HEK293 cells and increased the mRNA levels of *SEC24D*, a target gene of XBP1s. What is more, overexpression of RTP801 had the opposite effect, inhibiting the splicing of *XBP1* mRNA. These results agree with the work of Unlu *et al.* (55) who reported that the knockdown of the tRNA-LC antagonist CNP promoted XBP1 splicing and the transcription of *SEC24D*, whereas CNP upregulation inhibited *XBP1* splicing. These findings present the stress-responsive protein RTP801 as an important modulator of the mRNA ligase activity of the tRNA-LC *in vitro*. Interestingly, HSPC117 can be phosphorylated under stress conditions, which negatively affects *XBP1* mRNA splicing (59).

Remarkably, SEC24D regulates the formation and the cargo of the vesicles in the secretory pathway (60). Similarly, we have recently described that RTP801 mediates the production and the content of EVs (15). Vesicles from the secretory pathway can end up in multivesicular bodies (61), which fuse to the plasma membrane releasing EVs (62). Hence, we speculate that RTP801 might mediate the cargo of EVs via SEC24D. Nevertheless, *BDNF* expression was not affected by RTP801 levels, probably because HEK293 cells produce very low amounts of this neurotrophin

As aforementioned, XBP1s has an essential role in maintaining ER equilibrium. Indeed, an altered XBP1 pathway has been related with a variety of diseases, such as cardiovascular diseases (63,64), metabolic diseases, neurodegenerative diseases (65) and cancer (66). However, most of these findings occur in animal models, and do not perfectly reproduce the human pathophysiology. Therefore, here we wanted to study the status of XBP1 splicing in human postmortem samples. We found a drastic reduction in the splicing of XBP1s in the hippocampus of AD patients, which was accompanied by a significant increase in the levels of RTP801, as we had previously reported (8). In line with our results, Reinhardt *et al.* (67) found a significant reduction in the splicing of *XBP1* in the frontal cortex of AD patients, and a tendency to decrease in the hippocampus that did not reach significance because of the very low sample size. On the other hand, Hwan Lee *et al.* (68) described increased XBP1 splicing in the temporal cortex of AD patients compared to age-matched controls. It is speculated that XBP1 activation could happen at early time points in the pathology followed by a reduction as the disease progresses (69). As for the members of the tRNA splicing complex, we did not find any differences between AD patients and controls, which is in the line with our *in vitro* experiments. We also detected increased phosphorylation of eIF2α at serine 51 in AD hippocampi, in accordance with previous results studying P-eIF2α in the cortex (70,71) and hippocampus (72) of AD patients. These findings demonstrate the presence of an active UPR in AD patients and suggest that the impairment in *XBP1* splicing might be due to a localized defect in the splicing machinery rather than a general deficiency of the UPR. Interestingly, we also found that the protein levels of RTP801, XBP1s and P-eIF2α Ser51 were good predictors of the presence or absence of the disease. Therefore, their levels in blood and cerebrospinal fluid must be further studied as potential biomarkers of AD.

To complement our results in AD patients, we also investigated the splicing of *Xbp1* mRNA in a mouse model of the disease and whether it could be inhibited by RTP801. Previous literature showed that overexpression of XBP1s in *Drosophila* (73) and *Caenorhabditis elegans* (74) protected against Aβ- and tau-mediated neurotoxicity, respectively. As for mice, virus-mediated delivery of XBP1s in the hippocampus restored cognitive function and synaptic plasticity in the 5xFAD (47) and 3xTg-AD (48) models of AD. Moreover, Martínez *et al.* described that long-term potentiation and spatial memory were impaired in mice lacking XBP1 in the nervous system, and XBP1s overexpression in neurons enhanced long-term memory (46). They also found that XBP1s binds to the *Bdnf* promoter, and local expression of BDNF in the hippocampus of XBP1-deficient mice improved long-term memory. Thus, they propose that XBP1 splicing regulates cognition through the transcription of *BDNF* and other memory-related genes. Here, and as previously described (8), we did not detect increased levels of RTP801 protein in the hippocampus of 5xFAD mice although RTP801 mRNA levels were elevated. Moreover, we report that the silencing of RTP801 by a 25–30% specifically in hippocampal neurons causes increased splicing of *Xbp1* mRNA and increased levels of *Bdnf* mRNA in these animals.

We had previously described that RTP801 silencing in hippocampal neurons reduced neuroinflammation severity and restored cognitive deficits in 5xFAD (8) and R6/1 (9) mice. Taking all this into consideration, neuronal RTP801 silencing might be improving the AD phenotype through an increase in *Xbp1* mRNA splicing and BDNF production, among other mechanisms. It is worthy to mention that we chose to use this 6-month-old AD mouse model, instead of a one-cell-type primary culture, because in the 5xFAD mouse model the hippocampal architecture is preserved, the cognitive deficits are present and the neuroinflammatory response, due to astrocytes and microglia, is active. Hence, the use of this model was far more informative than a primary embryonic neuronal culture with no aging, no glia and no neuroinflammation present.

Besides its activity on *XBP1* splicing, the tRNA-LC performs the ligation of the tRNA exons during tRNA splicing. Using Hydro-tRNA-seq, we found that 5xFAD mice accumulate intron-containing pre-tRNAs compared to WT animals, which suggests an impairment in tRNA splicing in 5xFAD mice. Strikingly, this accumulation was not observed when the mature tRNAs were analyzed. Nonetheless, Kosmaczewski *et al.* (75) described that RtcB null worms (RtcB is the orthologue of HSPC117 in *C. elegans*) presented higher levels of intron-containing pre-tRNAs, with no apparent variation in the levels of the trimmed, ligated form (mature). Likewise, Lu *et al.* (76) found that the levels of unspliced tyrosine pre-tRNA were significantly higher in RtcB KO cells 3 days after depletion, while the levels of spliced tyrosine tRNA did not vary. Furthermore, our results are in accordance with those obtained by Sekulovski *et al.* (77), who studied tRNA splicing in patients with pontocerebellar hypoplasia (PCH) due to mutations in the splicing machinery, and concretely in the endonuclease complex. They found reduced splicing activity and accumulation of intron-containing pre-tRNAs in fibroblasts from PCH patients, analyzed by northern blot and by Hydro-tRNA-seq. Furthermore, in their work, global levels of mature tRNAs also remained unaffected. Here, we also report that RTP801 downregulation prevents the aberrant accumulation of intron-containing pre-tRNAs in 5xFAD mice. This reversion was observed in practically all the intron-containing pre-tRNA species, but not in those species without intron. For instance, tRNA-Arg-TCT-2–1 and tRNA-Arg-TCT-4–1 have the same anticodon (and thus are charged with the same amino acid) and have a 75.7% sequence homology. The main difference between them is the presence of an intron in the former. Nevertheless, tRNA-Arg-TCT-2–1 accumulates in 5xFAD mice, but tRNA-Arg-TCT-4–1 does not. This indicates that RTP801 specifically regulates tRNA splicing and thus the pool of intron-containing pre-tRNAs.

mt-tRNAs are not processed the same way that nuclear-encoded tRNAs. Indeed, mt-tRNA genes are transcribed as long polycistronic transcripts that contain multiple mt-tRNAs (78). Moreover, mt-tRNAs do not present introns and thus, are not spliced. The fact that we did not observe any difference in the abundance of mt-tRNAs between WT and 5xFAD mice suggests, once again, that only the processing of intron-containing tRNAs is altered in 5xFAD mice.

Finally, we found that the treatment of mouse hippocampal cultured neurons with the tRNA-enriched sRNA fraction from 5xFAD mice increases neuronal arborization with no changes in neuron viability. Specifically, we found an increase in the number of intermediate branches, which was accompanied by an augmented number of endpoints and increased tree length. Interestingly, increased spine density, branching and dendrite length was described in organotypic hippocampal cultures after Aβ treatment (79). Similarly, increased dendrite branching was found in a mouse model of AD (80). Indeed, it is speculated that excessive or aberrant synaptic plasticity might promote the development of AD (81). Conversely, dendritic degeneration has also been reported in AD (82,83). Altogether, our results suggest that the sRNAs found in the 60–100 nucleotide-sized fraction (mainly pre- and mature tRNAs, but also rRNAs and snoRNAs) derived from 5xFAD shCT mice (but not from 5xFAD shRTP801) influence the branching of the receiving neurons. This effect is probably due to the differential pool of intron-containing pre-tRNAs found in these mice, but it might also be due to the action of other sRNAs, or even to abnormally modified mature tRNAs.

All things considered, we present RTP801 as a new interactor of the tRNA-LC and a negative regulator of its mRNA and tRNA ligase activity. Our results suggest that in a context of AD, the protein levels of RTP801 rise and reduce the ligase activity of the tRNA-LC, in an attempt to cope with the stress conditions. In fact, the tRNA-LC is inhibited under oxidative stress conditions (54,84), possibly through a RTP801-mediated mechanism. As a result of this inhibition, the splicing of *XBP1* and the production of BDNF would be impaired, which might contribute to worsen the cognitive deficits and the inflammatory response in 5xFAD mice. Interestingly, both events are rescued by neuronal RTP801 silencing (8). The increase in RTP801 levels would also affect tRNA splicing, which would alter the pool of pre-tRNAs, with a specific accumulation of intron-containing species. Our proposed model regarding RTP801 activity agrees with the work of Pinto *et al.* (85), who described that ANGEL2, an antagonist of the tRNA-LC, affected the efficiency of tRNA and *XBP1* splicing.

The main limitation of our study is that we do not know the exact mechanism of action by which RTP801 interferes with the activity of the tRNA-LC. Although our data indicate that RTP801 interacts with the tRNA-LC and inhibits both its tRNA and mRNA ligase activity, we ignore the exact region of RTP801 that interacts with the members of complex. Mutating different regions of RTP801 protein and assessing the assembly and the activity of the tRNA-LC will be essential to better understand RTP801 inhibitory actions, and thus, to design potential AD therapies. Another question that remains unanswered is whether the inhibition of RTP801 in a different cell type than hippocampal neurons might also affect the tRNA pool and the XBP1s transcriptome, leading to an improvement in the 5xFAD phenotype. Finally, it will be worthy, although technically complicated, to investigate the potential detrimental effect of the intron-containing pre-tRNAs accumulated in 5xFAD mice, since they can generate microRNAs (86), nicked tRNAs (87), tRNA halves (88) or tRNA fragments with 2′,3′-cyclic phosphate ends (89). Altogether, our results place RTP801 as an important tRNA-LC modulator and support RTP801 as a promising pharmacological target in AD.

## Supplementary Material

gkae776_Supplemental_Files

## Data Availability

All data are available in the main text or the supplementary materials. Sequencing data (including raw data and normalized read counts) can be found at GEO (Gene Expression Omnibus, https://www.ncbi.nlm.nih.gov/geo/) with the following accession number: GSE267524.
