# Supplementary material for: RTP801 interacts with the tRNA ligase complex and dysregulates its RNA ligase activity in Alzheimer’s disease
Source: Nucleic Acids Res. 2024 Sep 12;52(18):11158–76. doi: 10.1093/nar/gkae776 (PMC11472047; doi:10.1093/nar/gkae776)
Supplement: gkae776_Supplemental_Files [file gkae776_supplemental_files.zip › Supplementary figures.pdf]

SUPPLEMENTARY FIGURES

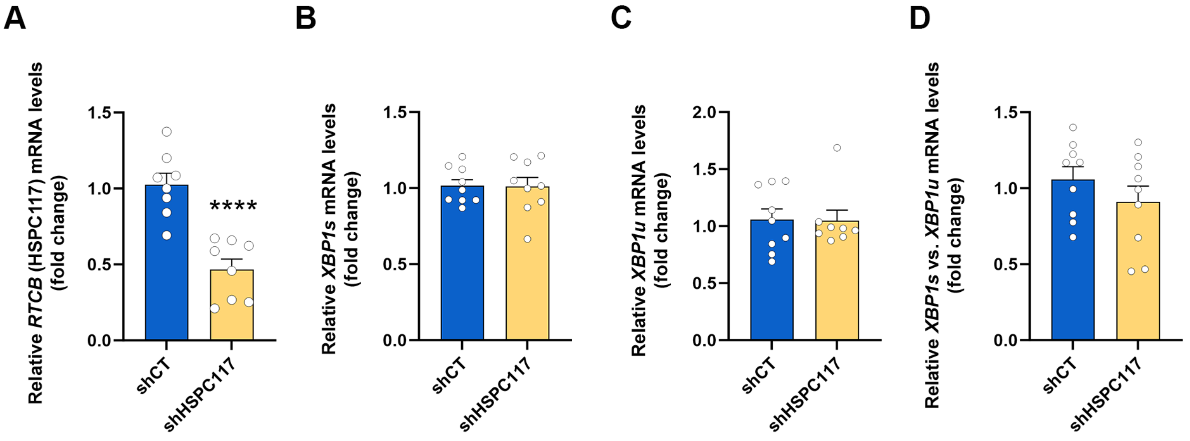

Supplementary Figure 1. HSPC117 downregulation does not impair *XBP1* splicing *in vitro*. HEK293 cells were transfected with shCT or shHSPC117 and 3 days later, RNA was extracted, retrotranscribed and RT-qPCR was performed. **(A-D)** RT-qPCR results for *RTCB* (HSPC117 coding gene) **(A)**, *XBP1s* **(B)**, *XBP1u* **(C)**, and *XBP1s/XBP1u* **(D)** (*RTCB*: $t_{14} = 5.493$, $p < 0.0001$; *XBP1s*: $t_{16} = 0.07421$, $p = 0.9418$; *XBP1u*: $U = 36$, $p > 0.9999$; *XBP1s/XBP1u*: $t_{16} = 1.107$, $p = 0.2846$). *ACTB* (β-actin) was used to normalize the expression of all genes. All data are analyzed with the unpaired two-tailed t-test. Data in panel **(C)** were analyzed with Mann-Whitney U test because values did not pass the normality test. All data are represented as mean ± SEM. Values represent technical replicates of 3 independent experiments. ****p < 0.0001.

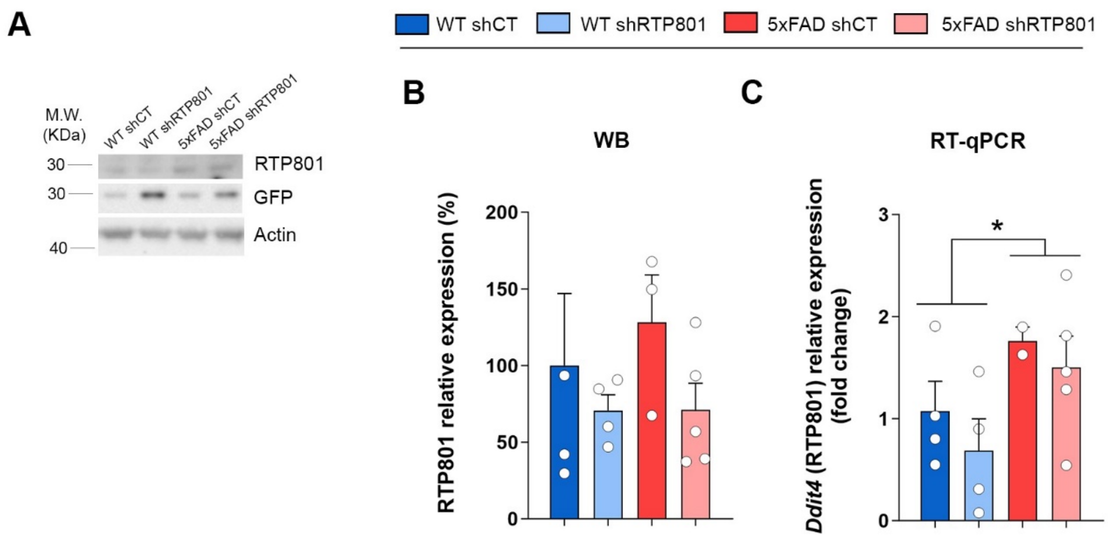

Supplementary Figure 2. Genetic inhibition of RTP801 levels in the dorsal hippocampus of 5xFAD mice. (A) WB for RTP801 and GFP as loading control for transduced neurons in the dorsal hippocampus of 7-month-old WT shCT, WT shRTP801, 5xFAD shCT, and 5xFAD shRTP801 groups of mice. **(B)** Densitometric quantification of RTP801 results as in **(A)** (Genotype effect: $F_{(1, 12)}$ = 0.2414, p = 0.6321, treatment effect: $F_{(1, 12)}$ = 2.204, p = 0.1634, interaction effect: $F_{(1, 12)}$ = 0.2286, p = 0.6412). **(C)** RT-qPCR results for *Ddit4* in the dorsal hippocampus of WT shCT, WT shRTP801, 5xFAD shCT, and 5xFAD shRTP801 groups of mice (Genotype effect: $F_{(1, 11)}$ = 5.033, p = 0.0464, treatment effect: $F_{(1, 11)}$ = 0.9219, p = 0.3576, interaction effect: $F_{(1, 11)}$ = 0.03395, p = 0.8572). *Hprt* was used to normalize the expression of *Ddit4*. Data are means ± SEM. In all comparisons two-way ANOVA with Tukey's *post hoc* test was performed. Each value represents one animal. *p < 0.05.

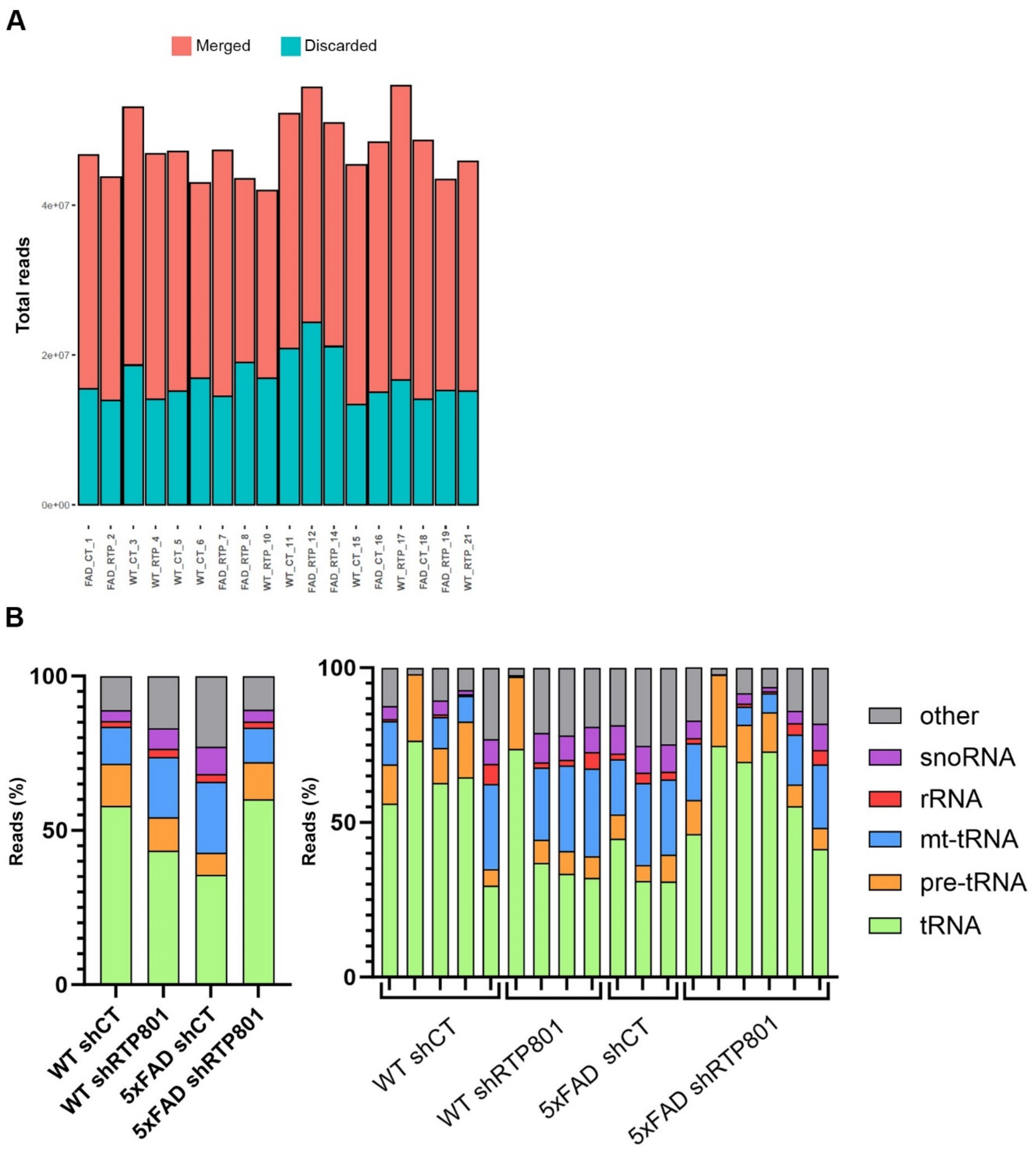

Supplementary Figure 3. Characterization of Hydro-tRNA-seq reads. (A) Sequencing read filtering (percentage of sequencing reads retained for read mapping). A filtering rate above 60% was considered satisfactory. **(B)** Read distribution of detected sRNA types, individually and grouped per condition. Most of the reads corresponded to mature tRNAs, but pre-tRNAs and mt-tRNAs were also present. rRNAs and snoRNAs (small nucleolar RNAs), were detected in a much lesser extent, as expected. For quality control, samples were considered satisfactory when >50% of the reads mapped tRNAs (either mature, precursor, or mitochondrial) and <35% of the reads mapped rRNAs or unannotated regions.

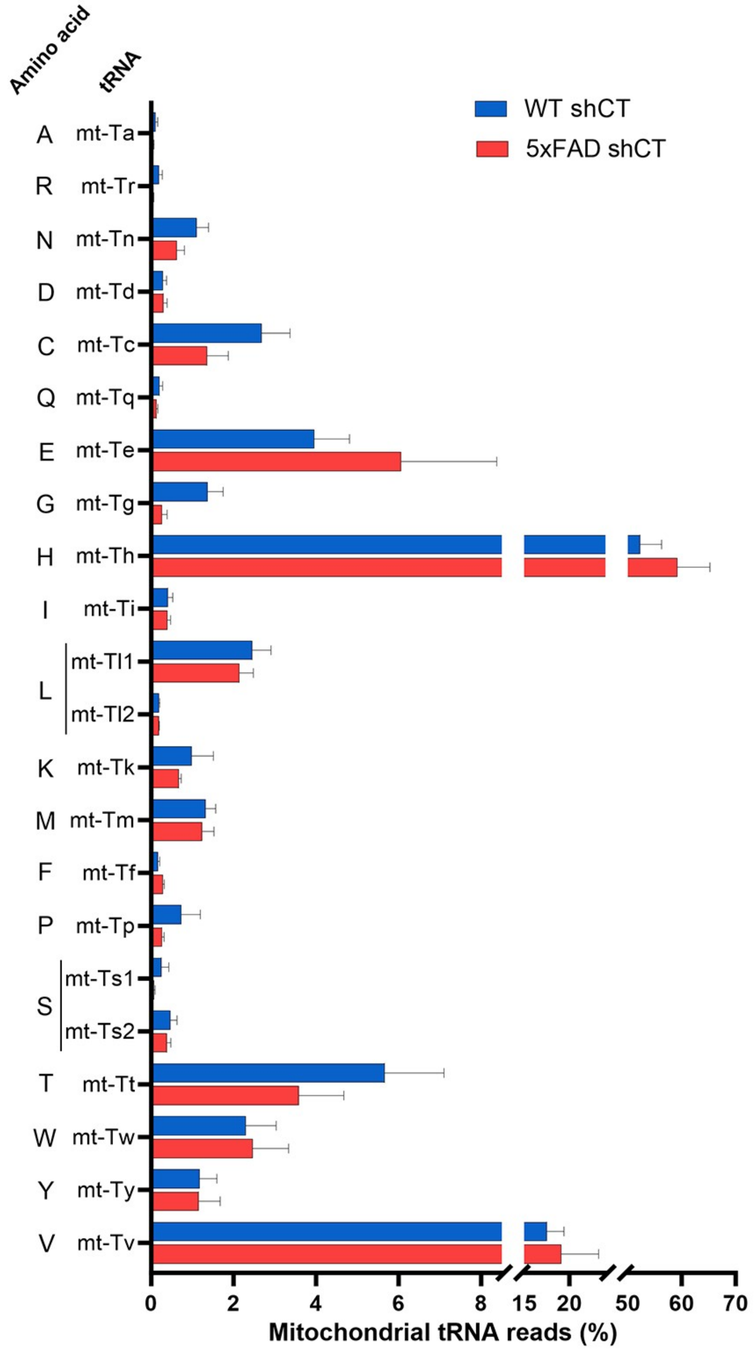

Supplementary Figure 4. The pool of mt-tRNAs is not altered in the dorsal hippocampus of 5xFAD mice. tRNAs were isolated from 7-month-old WT and 5xFAD mice and sequenced by Hydro-tRNA-seq. The percentage of normalized counts for mt-tRNAs is depicted, classified by amino acid. Data are means ± SEM. In all comparisons Student's t-test was performed.

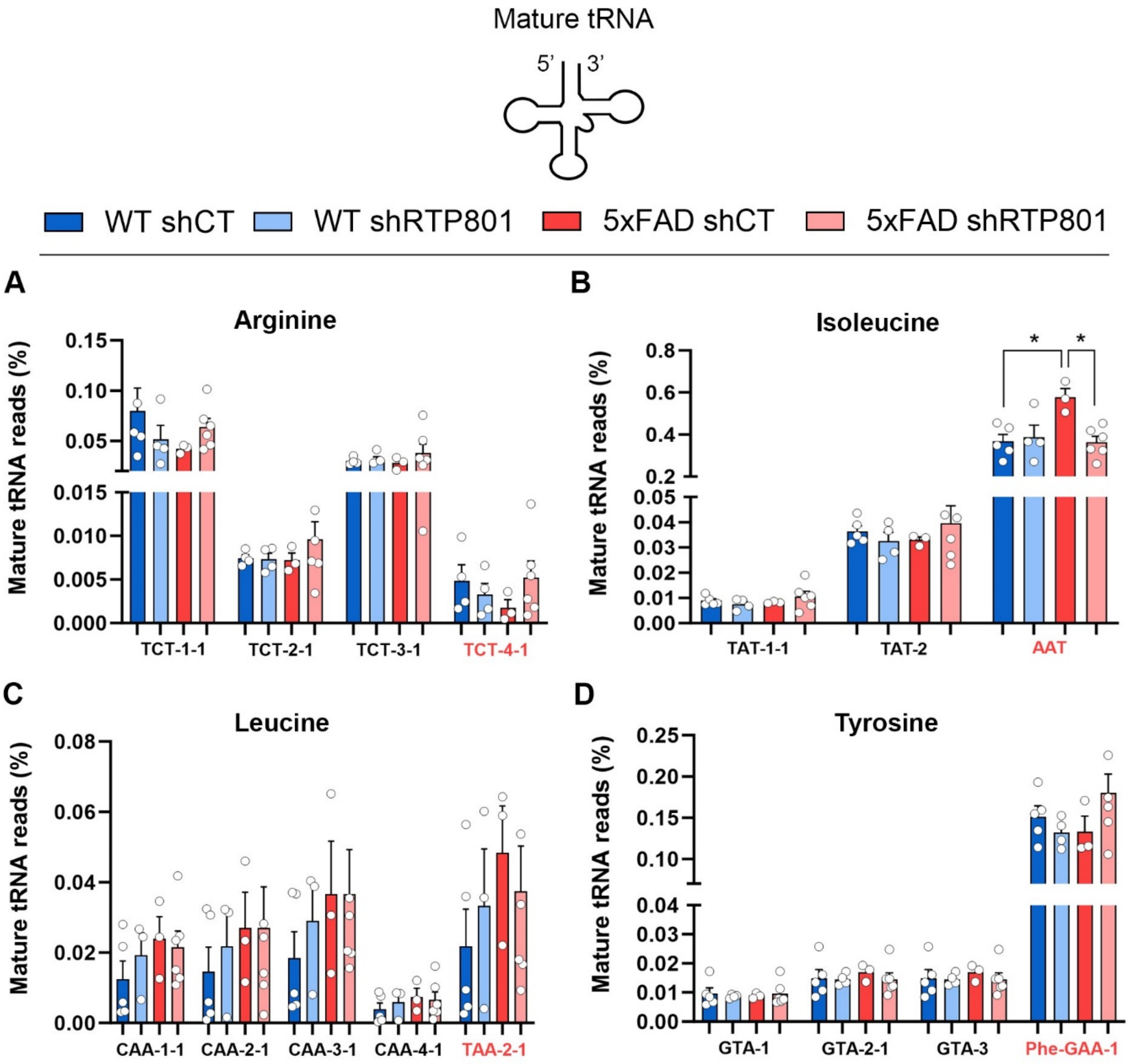

Supplementary Figure 5. RTP801 downregulation in hippocampal neurons does not affect the pool of mature tRNAs in 5xFAD mice. The percentage of normalized counts for mature tRNA is depicted, classified by amino acid and anticodon. Different tRNA species within an isodecoder family are represented. tRNA species in red do not have an intron (on its immature form) and are included as a control. Since all tyrosine-accepting pre-tRNAs have intron, tRNA-Phe-GAA-1 (also accepts an aromatic amino acid) was included as a control. Relative expression of tRNA-Arg-TCT **(A)**, tRNA-Ile-TAT **(B)**, tRNA-Leu-CAA **(C)**, and tRNA-Tyr-GTA **(D)** (tRNA-Ile-AAT: genotype effect: $F_{(1, 14)}$ = 5.136, p = 0.0398, treatment effect: $F_{(1, 14)}$ = 5.513, p = 0.0341, interaction effect: : $F_{(1, 14)}$ = 8.093, p = 0.0130). Data are means ± SEM. In all comparisons two-way ANOVA with Tukey's *post hoc* test was performed. Each value represents one animal. *p < 0.05.

Figure 2

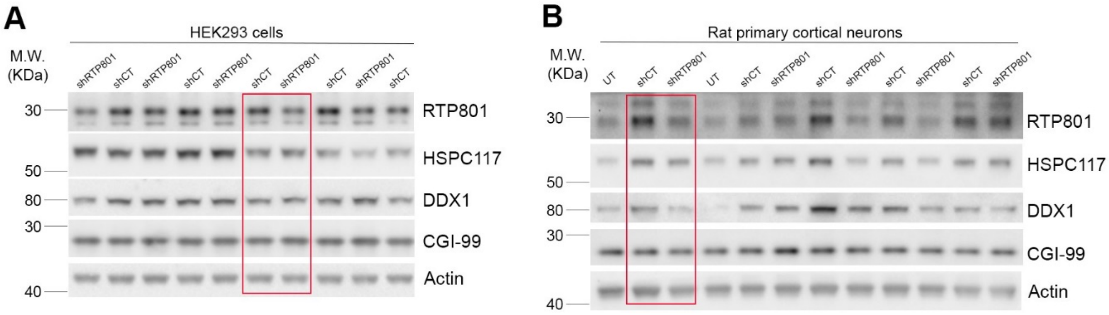

Figure 4

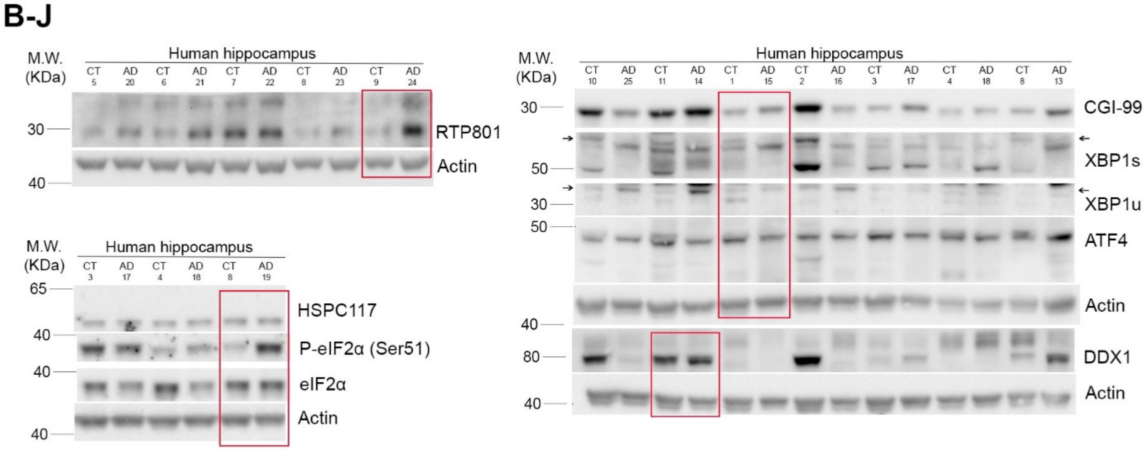

Supplementary Figure 2

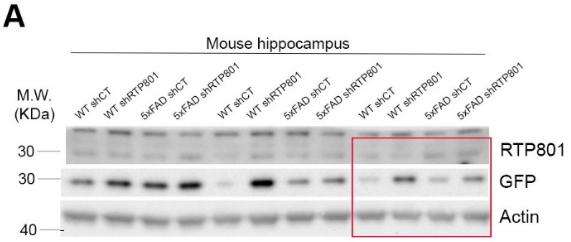

Supplementary Figure 6. Full-length blots for Figures 2, 4, and S2. Red rectangles indicate the part of each blot that was used in the panels of the figures.
