# Supplementary material for: RTP801 interacts with the tRNA ligase complex and dysregulates its RNA ligase activity in Alzheimer’s disease
Source: Nucleic Acids Res. 2024 Sep 12;52(18):11158–76. doi: 10.1093/nar/gkae776 (PMC11472047; doi:10.1093/nar/gkae776)
Supplement: gkae776_Supplemental_Files [file gkae776_supplemental_files.zip › Supplementary tables NAR.pdf]

Supplementary Table 1

Clinical diagnosis	Patient	Braak stage	Thal stage	Sex	Age (years)	PMD (hh:mm)
CT	1	II	5	F	97	7:20
	2	II	4	F	93	5:30
	3	II	3	M	86	7:35
	4	II	2	F	88	24:00
	5	II	3	M	64	10:00
	6	0	1	M	83	13:00
	7	III	0	M	86	7:25
	8	I-II	1	M	78	6:00
	9	0	0	M	76	11:30
	10	III	3	F	90	13:40
	11	II	0	F	83	7:30
	12	II	5	F	83	7:33
AD	13	VI	5	F	84	11:00
	14	VI	5	M	78	7:20
	15	VI	5	F	90	5:30
	16	V	4	F	83	10:45
	17	VI	5	F	78	11:30
	18	VI	5	F	88	13:30
	19	VI	5	F	82	16:45
	20	V-VI	5	M	77	7:30
	21	VI	5	M	82	4:30
	22	VI	5	F	64	5:30
	23	VI	5	F	74	6:30
	24	V	4	M	76	6:00
	25	VI	5	F	85	12:00
	26	VI	4	F	80	15:00
	27	V	3	M	86	17:30
	28	V	5	F	85	16:00

Supplementary Table 1. Human postmortem hippocampal samples. PMD, postmortem delay; M, male; F, female.

Supplementary Table 2

Empty vector and characteristics	Construct	Characteristics
**pLL3.7**<br>Purchased from Addgene. Empty backbone for expression of shRNA under the U6 promoter. Confers ampicillin resistance and expresses a CMV-eGFP reporter to monitor expression.	**pLL3.7-shCT**	Validated scrambled control sequence 5'-GTGCGTTGCTAGTACCAAC-3' for human, rat, and mouse [7].
	**pLL3.7-shRTP801**	Validated sequence 5'-AAGACTCCTCATACCTGGATG-3' targeting human, rat, and mouse RTP801 [7].
**rAAV2/8**<br>Provided by the Viral Vector Production Unit (Universitat Autònoma de Barcelona). Expresses the shRNA under the H1 promoter and eGFP under the control of RSV promoter.	**rAAV2/8-shCT**	Validated scrambled control sequence 5'-GTGCGTTGCTAGTACCAAC-3' for mouse [8], [9].
	**rAAV2/8-shRTP801**	Validated sequence 5'-AAGACTCCTCATACCTGGATG-3' for mouse RTP801 [8], [9].
**pRP**<br>Purchased from VectorBuilder. Empty backbone for expression of shRNA under the U6 promoter. Confers ampicillin resistance and expresses an hPGK-mCherry reporter to monitor expression.	**pRP-shCT**	Scrambled control sequence 5'-CCTAAGGTTAAGTCGCCCTCG-3' for human and mouse.
	**pRP-shHSPC117**	Sequence 5'-CAATGAATGCCAAAGACTTGG-3' for human, rat, and mouse HSPC117.
**FUGWm**<br>Purchased from Addgene. Empty backbone for gene overexpression under the CMV promoter. Confers ampicillin resistance and expresses an UbC-eGFP reporter to monitor expression.	**FUGWm-eGFP**	The original construct (pCMS-eGFP) was designed and validated in [7]. It was then subcloned to obtain FUGWm-eGFP, which was validated in [15].
	**FUGWm-eGFP-RTP801**	The original construct (pCMS-eGFP-RTP801) was designed and validated in [7]. It was then subcloned to obtain FUGWm-eGFP-RTP801, which was validated in [15].

Supplementary Table 2. List of plasmids used. shRNA = short hairpin RNA; CMV = cytomegalovirus; eGFP = enhanced green fluorescent protein; rAAV2/8 = recombinant adeno-associated virus serotypes 2/8; RSV = Rous sarcoma virus; UbC = ubiquitin C. Plasmids validated in [7], [8], [9], [15].

Supplementary Table 3

Antibody	Host	Dilution	Source
DDX1 (for IP)	Rabbit	1:1000	Bethyl, #A300-512A
DDX1	Mouse	1:500	Santa Cruz Biotechnology, #sc-271438
RTP801	Rabbit	1:500	Proteintech, #10638-1-AP
HSPC117	Mouse	1:1000	Santa Cruz Biotechnology, #sc-393966
CGI-99	Rabbit	1:500	Proteintech, #19848-1-AP
HRP-β-actin	Mouse	1:100,000	Merck, #A3854
XBP1	Rabbit	1:500	Abcam, #ab37152
P-eIF2α Ser51	Rabbit	1:500	Cell Signaling Technologies, #9721
eIF2α	Rabbit	1:500	Cell Signaling Technologies, #9722
ATF4	Rabbit	1:500	Proteintech, #10835-1-AP
GFP	Rabbit	1:800	Cell Signaling Technologies, #2956

Supplementary Table 3. Antibodies used for WB. IP = immunoprecipitation.

Supplementary Table 4

Sample	Condition	RIN
1	5xFAD shCT	8.1
2	5xFAD shRTP801	8.2
3	WT shCT	8.4
4	WT shRTP801	8.5
5	WT shCT	8.4
6	WT shCT	8.5
7	5xFAD shRTP801	8.1
8	5xFAD shRTP801	8.1
10	WT shRTP801	8
11	WT shCT	8.4
12	5xFAD shRTP801	8.5
14	5xFAD shRTP801	8.7
15	WT shCT	8.6
16	5xFAD shCT	8.4
17	WT shRTP801	8.5
18	5xFAD shCT	8.3
19	5xFAD shRTP801	8.5
21	WT shRTP801	8.9

Supplementary Table 4. RNA integrity (RIN) of the mouse hippocampal samples used for Hydro-tRNA-seq.

Supplementary Table 5

Target gene	Specie	Primer	Sequence (5'- 3')	Amplicon size (nt)	Source
DDIT4 (RTP801)	*Hs*	Fw	TTTGGGACCGCTTCTCGTC	388	Own design
		Rv	CGCAGTAGTTCTTTGCCCAC		
	Mm	Fw	GCCTAGCCTTTGGGATCGTT	520	
		Rv	CAGGGTCAACTGAAAGGTGG		
XBP1 (spliced)	*Hs, Mm*	Fw	GAGTCCGCAGCAGGTG	150	[19]
		Rv	GGAAGGGCATTTGAAGAACA		
XBP1 (unspliced)	*Hs, Mm*	Fw	ACTACGTGCACCTCTGCAG	159	
		Rv	GGAAGGGCATTTGAAGAACA		
BDNF (proBDNF and mature)	*Hs, Mm*	Fw	GGCGGCAGATAAAAAGACTG	202	Own design
		Rv	TACCCAGTCGTATGTTCGG		
SEC24D	*Hs*	Fw	TGGACCAGTCAGATGCAACAGG	155	Origene (#HP211178)
		Rv	GGACCACATTGGAAGAAGACTGG		
Kalrn (Kalirin isoform 7)	*Mm*	Fw	GATACCATATCCATTGCCTCCA GGACC	127	[77]
		Rv	CCAGGCTGCGCGCTAAACGTA AG		
RTCB (HSPC117)	*Hs*	Fw	GAAGGAGCAACTTGCCCAAGC T	161	Own design
		Rv	AGTGCTCCTTGTCTTCAGCCCA		
ACTB (Actin)	*Hs*	Fw	TTGCCGACAGGATGCAGAAGGA	129	[78]
		Rv	AGGTGGACAGCGAGGCCAGGAT		
Hprt1	*Mm*	Fw	TGTTGTTGGATATGCCCTTG	259	[79]
		Rv	AATGTCAGTTGCTGCGTCC		

Supplementary Table 5. Primers used for RT-qPCR. *Hs = Homo sapiens; Mm = Mus musculus*; Fw = Forward; Rv = Reverse. Primers obtained from [19], [87], [88], [89].

Supplementary Table 6

tRNAs with intron	
Arg-TCT-1-1	Ile-TAT-2-3
Arg-TCT-2-1	Tyr-GTA-1-1
Arg-TCT-3-1	Tyr-GTA-1-2
Arg-TCT-5-1	Tyr-GTA-1-3
Leu-CAA-1-1	Tyr-GTA-1-4
Leu-CAA-2-1	Tyr-GTA-1-5
Leu-CAA-3-1	Tyr-GTA-2-1
Leu-CAA-4-1	Tyr-GTA-3-1
Ile-TAT-1-1	Tyr-GTA-3-2
Ile-TAT-2-1	Tyr-GTA-4-1
Ile-TAT-2-2	Tyr-GTA-5-1

Supplementary Table 6. List of the 22 tRNAs with intron found in mice according to GtRNAdb.
tRNAs in red were not detected by Hydro-tRNA-seq.

Supplementary table 7

Antibody	Host	Dilution	Source
MAP2	Mouse	1:500	Abcam, #ab11268
Cleaved Caspase 3	Rabbit	1:200	Cell Signaling Technologies, #9661
Alexa Fluor™ 488 anti-mouse	Goat	1:500	Thermo Fisher Scientific, #A11017
Alexa Fluor™ 555 anti-rabbit	Goat	1:500	Thermo Fisher Scientific, # A21430

Supplementary table 7: Antibodies used for immunofluorescence.
